# Tomato Prosystemin Is Much More than a Simple Systemin Precursor

**DOI:** 10.3390/biology11010124

**Published:** 2022-01-13

**Authors:** Donata Molisso, Mariangela Coppola, Martina Buonanno, Ilaria Di Lelio, Simona Maria Monti, Chiara Melchiorre, Angela Amoresano, Giandomenico Corrado, John Paul Delano-Frier, Andrea Becchimanzi, Francesco Pennacchio, Rosa Rao

**Affiliations:** 1 Department of Agricultural Sciences, University of Naples Federico II, Via Università 100, 80055 Naples, Italy; donata.molisso@unina.it (D.M.); mariangela.coppola@unina.it (M.C.); ilaria.dilelio@unina.it (I.D.L.); giacorra@unina.it (G.C.); andrea.becchimanzi@unina.it (A.B.); f.pennacchio@unina.it (F.P.); 2 Materias s.r.l., Corso N. Protopisani 50, 80146 Naples, Italy; 3 Istituto di Biostrutture e Bioimmagini-CNR, Via Mezzocannone 16, 80134 Naples, Italy; martina.buonanno@cnr.it; 4 Department of Chemical Sciences, University of Naples Federico II, Complesso Universitario di Monte Sant’Angelo, Via Cinthia 4, 80126 Naples, Italy; chiara.melchiorre@unina.it (C.M.); angela.amoresano@unina.it (A.A.); 5 Center for Research and Advanced Studies (CINVESTAV) Irapuato, Department of Biochemistry and Biotechnology, Km. 9.6 Libramiento Norte Carretera Irapuato-León, Irapuato 36500, Mexico; john.delano@cinvestav.mx; 6 Interuniversity Center for Studies on Bioinspired Agro-Environmental Technology (BAT Center), University of Naples Federico II, Via Università 100, 80055 Naples, Italy

**Keywords:** systemin, transgenic plants, transcriptomics, peptide direct delivery, intrinsically disordered proteins, plant defense, plant pests, oligogalacturonides

## Abstract

**Simple Summary:**

Prosystemin is a 200 amino acid precursor that releases, upon wounding and biotic attacks, an 18 amino acid peptide called Systemin. This peptide was traditionally considered as the principal actor of the resistance of tomato plants induced by triggering multiple defense pathways in response to a wide range of biotic/abiotic stress agents. Recent findings from our group discovered the disordered structure of Prosystemin that promotes the binding of different molecular partners and the possible activation of multiple stress-related pathways. All of our recent findings suggest that Prosystemin could be more than a simple precursor of Systemin peptide. Indeed, we hypothesized that it contains other sequences able to activate multiple stress-related responses. To verify this hypothesis, we produced a truncated Prosystemin protein deprived of the Systemin peptide and the relative deleted gene. Experiments with transgenic tomato plants overexpressing the truncated Prosystemin and with plants exogenously treated with the recombinant truncated protein demonstrated that both transgenic and treated plants modulated the expression of defense-related genes and were protected against a noctuid moth and a fungal pathogen. Taken together, our results demonstrated that Prosystemin is not a mere scaffold of Systemin, but itself contains other biologically active regions.

**Abstract:**

Systemin (Sys) is an octadecapeptide, which upon wounding, is released from the carboxy terminus of its precursor, Prosystemin (ProSys), to promote plant defenses. Recent findings on the disordered structure of ProSys prompted us to investigate a putative biological role of the whole precursor deprived of the Sys peptide. We produced transgenic tomato plants expressing a truncated ProSys gene in which the exon coding for Sys was removed and compared their defense response with that induced by the exogenous application of the recombinant truncated ProSys (ProSys_(1-178),_ the Prosystemin sequence devoid of Sys region). By combining protein structure analyses, transcriptomic analysis, gene expression profiling and bioassays with different pests, we demonstrate that truncated ProSys promotes defense barriers in tomato plants through a hormone-independent defense pathway, likely associated with the production of oligogalacturonides (OGs). Both transgenic and plants treated with the recombinant protein showed the modulation of the expression of genes linked with defense responses and resulted in protection against the lepidopteran pest *Spodoptera littoralis* and the fungus *Botrytis cinerea*. Our results suggest that the overall function of the wild-type ProSys is more complex than previously shown, as it might activate at least two tomato defense pathways: the well-known Sys-dependent pathway connected with the induction of jasmonic acid biosynthesis and the successive activation of a set of defense-related genes, and the ProSys_(1-178)_-dependent pathway associated with OGs production leading to the OGs mediate plant immunity.

## 1. Introduction

In-depth studies on plant defense responses in Solanaceae have shown the important role of signaling peptides associated with tissue wounding and insect herbivory [1,2,3]. These peptides include tomato Systemin (Sys), an octadecapeptide released upon wounding from Prosystemin (ProSys), a precursor protein of 200 amino acids, through a poorly defined pathway, apparently mediated by phytaspases [4,5]. Sys promotes long-distance defense responses by amplifying the jasmonate signaling pathway, which appears to be central to systemic defense signaling [6]. Other members of the same family of defensive peptides are hydroxyproline-rich systemins (HypSys), which are also released from a larger precursor [7]. Although these peptides are structurally unrelated to Sys, they took their name from their systemin-like function [8]. ProSys and HypSys work cooperatively in the regulation of tomato defense responses [9].

The modulation of direct and indirect defenses against herbivorous insects by Sys has been widely characterized [3,10,11]. The constitutive expression of the ProSys gene in tomato plants triggers the increase of protease inhibitors (PIs) and other defensive compounds conferring resistance to chewing and sucking insects, phytopathogenic fungi and salt stress [1,3,5,12,13,14,15]. In addition, these transgenic plants are characterized by an increased level of indirect defense barriers compared to untransformed controls, showing a higher level of attractiveness towards natural enemies of phytophagous insects [10,11,16]. This is probably the consequence of the consistent transcriptomic reprogramming observed in transgenics, resulting in more than 500 differentially expressed genes [12]. The analysis of these genes showed that ProSys overexpression, on the one hand, reduces the expression of genes related to carbohydrate metabolism and, on the other hand, promotes the expression of a series of defense genes regulated by different signaling pathways, thus cross-modulating growth and defense pathways [12]. The Sys peptide is considered to be solely responsible for the biological activity in tomato, as suggested by the increased production of PIs upon Sys application on wounded or intact stems and leaves [2,6,17,18,19]. Thus, a single peptide appears to trigger multiple defense pathways in response to a wide range of stress agents [10]. 

The mechanism underlying such a large “anti-stress” capacity, associated with a single peptide, is difficult to understand from a functional point of view. Perhaps Sys “is not alone” in the activation of tomato defense responses as suggested by the structural features of ProSys protein. In fact, it was very recently demonstrated that ProSys is an intrinsically disordered (ID) protein [20] without a stable or ordered three-dimensional structure. ID proteins (IDPs) play a central role in regulating the transduction pathways of various signals, including the “defense signal”, in addition to other crucial cellular processes, such as the regulation of transcription and translation [21]. It has been proposed that the plasticity of IDPs helps sessile organisms, such as plants, in establishing complex networks in response to the exposure to a myriad of both biotic and abiotic stress agents, from which they cannot move away to prevent damage [22]. The characteristic flexibility of IDPs allows them to assume a number of conformations able to target multiple molecular partners [23]. Therefore, based upon the above assumptions, we postulate that ProSys can be more than a simple precursor of Sys and that it integrates additional functions likely activating multiple stress-related pathways upon interacting with different molecular partners. This hypothesis is also supported by the altered proteomic profile and increased resistance against *Botrytis cinerea* observed in tobacco transgenic plants constitutively expressing the truncated ProSys, which lacks the Sys-encoding exon [24]. 

Here we provide several pieces of evidence in support of this intriguing hypothesis. A wealth of molecular and functional data on transgenic tomato plants, constitutively expressing the truncated ProSys cDNA, and on their interactions with *S. littoralis* and *B. cinerea*, have been gathered, showing a multifaceted enhanced resistance against these biotic stress agents. Results have been corroborated by exogenous treatments with a recombinant ProSys deprived of the Sys region (hereafter referred to as ProSys_(1-178)_ protein), which provides further direct evidence in support of the multifunctional role of ProSys in orchestrating the defense response of tomato plants.

## 2. Materials and Methods

### 2.1. Plant Material and Growth Condition

Tomato seeds (*Solanum lycopersicum* L. cultivar “Red Setter”) were surface sterilized by soaking in 70% ethanol for 2 min, rinsed, washed with 2% sodium hypochlorite for 10 min and then rinsed at least five times with sterile water. Seeds were then germinated in Petri dishes on wet sterile paper and kept in the dark for 3 days in a growth chamber at 24 ± 1 °C and 60% of relative humidity (RH). Upon roots emergence, plantlets were transferred to a polystyrene tray with barren sterile S-type substrate (FloraGard; Oldenburg, Germany) in a growth chamber at 26 ± 1 °C and 60% RH with an 18:6 h light/dark photoperiod. After 2 weeks, the plants were transferred into 9 cm diameter pots filled with a sterile soil mixture using the same growth conditions.

### 2.2. Tomato Transgenic Plants Production and Analysis

The pPRO binary vector [24], containing cDNA of the coding region (CDS) of the tomato Prosystemin lacking the last exon coding for the Sys peptide plus a truncated form of the 5’ and 3′ untranslated region (UTR), 15 bp of the 5′-UTR and 103 bp of the 3′-UTR, under the control of the CaMV 35S RNA promoter and the pea rbcS terminator, was used for the genetic transformation of *S. lycopersicum* L. “Red Setter” as previously described [12,24]. Putative transformants, selected on kanamycin (50 µg/mL), were analyzed by PCR to detect the transgene, as already described [12]. The isolation of total RNA from leaves of four-week-old plants grown in sterile soil, the synthesis of the first strand of cDNA and Real-Time RT-PCR were performed as previously reported [25]. Transgenic plants are hereafter referred to as ProSys_(1-178)_ plants. According to the transgene expression levels, 2 plants of the T_0_ generation were reproduced up to T_4_ generation to select genotypes homozygous for a single copy of the transgene. These lines are indicated as lines 1 and 2.

### 2.3. Molecular Cloning, Expression, and Purification of ProSys_(1-178)_

ProSys_(1-178)_ was obtained after PCR amplification of ProSys cDNA (GenBank: AAA34184.1) with site-specific synthetic primers (Table S1) and was cloned in the pETM11 protein expression vector (a kind gift from EMBL, Heidelberg). The generated plasmid was checked by DNA sequencing and appropriate digestion with restriction enzymes. The recombinant product was expressed in *E. coli*, BL21(DE3) strain, induced with 1 mM isopropyl-β-D-1-tiogalattopiranoside (IPTG) for 16 h at 22 °C in 2-YT broth. Cells were harvested by centrifugation and re-suspended as previously reported [20]. Cells were then disrupted by sonication, and after centrifugation (30 min at 30,000× *g* at 4 °C), the supernatant (soluble fraction) was purified by FPLC on a 1 mL HisTrap FF column (GE Healthcare; Milan, Italy) by stepwise elution, according to the manufacturer’s instruction. Fractions containing ProSys_(1-178)_ protein were dialyzed in PBS 1X (Phosphate buffer saline, 10 mM phosphate, 140 mM NaCl, 2.7 mM KCl, pH 7.4), 100 µM PMSF, 1 mM DTT pH 8.0 using a dialysis membrane with a molecular weight cut-off (MWCO) of 3500 Da for 16 h at 4 °C. ProSys_(1-178)_ was finally purified in PBS 1X by size exclusion chromatography (SEC) on a Superdex 75 10/300 HP column (GE Healthcare; Milan, Italy). A molecular weight calibration curve was obtained using the following standards (Sigma-Aldrich, St. Louis, MO, USA): horse cytochrome c (Cit c, 12.4 kDa), chicken ovalbumin (Ova, 45 kDa), bovine serum albumin (BSA, 66 kDa), carbonic anhydrase from bovine erythrocytes (CA, 29 kDa), recombinant carbonic anhydrase XIV (CA XIV, 37 kDa, homemade). The purity level of the recombinant protein was assessed by 15% SDS-PAGE using Bio Rad Precision Plus Protein All Blue Standards (10–250 kDa) as molecular weight ladder (Bio-Rad; Hercules, CA, USA).

### 2.4. LC-ESI-MS, Circular Dichroism and Light Scattering Analyses

LC-ESI-MS and circular dichroism (CD) spectra were performed as previously described [20,26] to confirm protein identity and behavior. SEC-MALS-QELS analysis of ProSys_(1-178)_ was performed at 0.5 mL/min in PBS 1×, 100 µM PMSF, 1 mM DTT pH 8.0 on an SEC 2000 column (Phenomenex; Torrance, CA, USA) linked to an FPLC ÄKTA coupled to a light scattering detector (mini-DAWN TREO, Wyatt Technology; Santa Barbara, CA, USA) and to a refractive index detector (Shodex RI-101; Showa Denko, Tokyo, Japan). Collected data were processed using the ASTRA 5.3.4.14 software (Wyatt Technologies Corporation).

### 2.5. Plant Treatments with ProSys_(1-178)_

Fifteen spots of 2 µL of 100 pM ProSys_(1-178)_ solution were carefully placed on the abaxial surface of fully expanded healthy leaves of four-week-old tomato plants (a mock treatment with buffer was used as control). The treated leaves were collected 6 and 24 h after ProSys_(1-178)_ application for molecular investigations and for bioassays unless otherwise indicated.

### 2.6. OGs Extraction by Chelating Agent

The OGs extraction protocol was a modified version of the protocol described in [27]. For each sample, about 50 mg of crushed fresh leaves were re-suspended in 1 mL of 70% ethanol, centrifuged for 15 min at 14,000 rpm. The pellet was washed twice with a chloroform:methanol (1:1, vol/vol) mixture, vortexed and centrifuged at 14,000 rpm for 15 min. The pellet was washed twice with acetone, centrifuged at 14,000 rpm for 15 min and dried under vacuum. The pellet was re-suspended in 200 μL of ultrapure water and kept overnight at 4 °C on a wheel. After centrifugation for 30 min at 14,000 rpm, the supernatant was discarded and the pellet was re-suspended in 200 μL chelating agent solution (ChA = 50 mM EDTA dissolved in 1 M NaOH) and incubated overnight at 4 °C on a wheel. After centrifugation for 30 min at 14,000 rpm, the supernatant containing the chelating agent soluble fraction (ChASF) was recovered. Oligogalacturonides (OGs) contained in the ChASF were precipitated with 1 mL of 80% ethanol at −20 °C overnight. Pellets obtained by centrifugation at 14,000 rpm for 30 min were washed twice with 80% ethanol and dried under vacuum. OGs were dissolved in 100 μL ultrapure water and subjected to MALDI-TOF analysis.

### 2.7. MALDI-TOF (Matrix Assisted Laser Desorption Ionization-Time of Flight) Mass Spectrometry

One microliter of extracted OGs was mixed with 4 μL of MALDI matrix (50 mg/mL of PA/HPA/AC 18:1:1) and 1 μL of the mix was spotted on the MALDI plat. Mass spectra were recorded on a 5800 plus MALDI TOF-TOF mass spectrometer (ABI SCIEX) equipped with a reflectron analyzer and used in delayed extraction mode with 4000 Series Explorer v3.5 software. MALDI-MS data were acquired over the mass range of 100−3000 *m*/*z* in the positive ion mode. Each spectrum represents the sum of 400 laser pulses from randomly chosen spots per sample position. Three biological replicates for the two transgenic lines and for ProSys_(1-178)_-treated plants were used for the analysis.

### 2.8. Bioassays

#### 2.8.1. Herbivory by *S. littoralis* Larvae

The impact of the experimental plants on *S. littoralis* larvae (Lepidoptera, Noctuidae) was assessed as previously described [17], starting from a larval population reared on an artificial diet [28] at 25 ± 1 °C, 70 ± 5% RH under a 16:8 h light/ dark photoperiod. The feeding bioassays were performed under the same environmental conditions, in polystyrene rearing trays (RT32W, Frontier Agricultural Sciences; Newark, Germany), bottom-lined with 3 mL of 1.5% agar (*w*/*v*) on which 150 newly hatched larvae were deposited, in groups of 50 individuals, on leaf disks and allowed to develop fully to the 2^nd^ instar. The rearing wells were closed by perforated plastic lids (RTCV4, Frontier Agricultural Sciences; Newark, Germany). Soon after molting to the 3^rd^ instar, 32 larvae, for each experimental condition, were singly transferred into new trays prepared as above and were daily offered fresh leaf disks of uniform size (initially of 1 cm^2^; later, disks of 2, 3, 4 and 5 cm^2^ were offered to meet the increasing nutritional needs of the larvae), obtained from sub-apical leaves of 4-week-old plants. For the experiments performed with transgenic plants, larvae were fed with tomato leaf disks of 2 transgenic plant lines 1 and 2 and of untransformed control plants, while for the experiments with plants treated with 100 pM ProSys_(1-178)_, leaf discs of treated and control plants were supplied daily to the larvae, 6 h after plant treatment.

The survival rate was assessed daily, while the larval weights were recorded every 5 days for the ProSys_(1-178)_ transgenic plants experiments and every two days for the ProSys_(1-178)_-treated plant experiments. These parameters were recorded until pupation.

#### 2.8.2. Infection by the Necrotrophic Fungus *B. cinerea*

For this bioassay, 4-week-old ProSys_(1-178)_ transgenic plants were inoculated with fungal spores as previously described [29]. Briefly, spores of *B. cinerea* were suspended in sterile distilled water, filtered through sterile Kimwipes (Kimberly-Clark; Dallas, TX, USA) and adjusted to a concentration of 1 × 10^6^ spores/mL. A total of 10 µL of the spore suspension was applied between the leaf veins, using four different inoculation points per leaf. Five plants for each of lines 1 and 2 were used. Lesion’s diameters were measured at different time points (1, 3, 5 and 8 days post inoculum) using a digital caliper (Neiko 01407A; Neiko Tools, Taiwan, China). For each sample, 2 technical replicates were used.

The assay was also performed on detached leaves exogenously supplied with ProSys_(1-178)_. For this purpose, leaves of four-week-old plants were harvested and treated with 100 pM recombinant protein or control buffer and, after 6 h, a spore suspension of *B. cinerea* was applied. Necroses were measured as reported above. Three leaves from three different plants per each treatment were used.

### 2.9. Two-Color Microarray-Based Gene Expression Analysis

Total RNA was extracted from leaves using the Plant RNeasy mini kit (Qiagen; Hilden, Germany), according to the manufacturer’s protocol. RNA quantification and quality control were carried out with the 2100 Bioanalyzer system (Agilent Technologies: Santa Clara, CA, USA). Samples with a 260/280 nm absorbance ratio > 1.8 and a 260/230 nm absorbance ratio > 2 were labeled and hybridized to the Tomato Gene Expression Microarray 4 × 44 K (Agilent Technologies) as previously described [12]. Experiments were run in triplicate for each biological condition. Upon scanning and image data processing, raw data were analyzed using as already reported techniques [12]. Differentially expressed RNAs were identified after filtering by the Benjamini and Hochberg False Discovery Rate (*p* < 0.05) and a minimum of a 2-fold variation in expression compared to the untransformed controls. Differentially expressed genes (DEGs) functional annotation was carried out by sequence analysis using the Blast2GO software [30]. Mapping of enzymatic activities into molecular pathways was acquired from the Kyoto Encyclopedia Gene and Genomes (KEGG) database.

Microarray data were validated by Real-Time RT-PCR targeting 7 DEGs. The expression analysis was extended to the other 2 transgenic genotypes (ProSys_(1-178)_ lines 1 and 2).

### 2.10. Relative Quantification of Gene Expression

Expression analyses were carried out by Real-Time RT-PCR using Rotor Gene 6000 (Corbett Research; Sydney, Australia). The isolation of total RNA from leaves of 4-week-old plants grown in sterile soil, the synthesis of the first-strand cDNA and Real-Time RT-PCR were performed as previously reported [25]. Two technical replicates for each of the three biological replicates per sample were used. The housekeeping gene EF-1α was used as the reference gene. Relative quantification of gene expression was carried out using the 2^−ΔΔCt^ method [31]. Primers and their main features are described in Table S1.

### 2.11. Statistical Analysis

Relative quantification of the transcript abundance was compared by Student’s *t*-test when tests were compared to controls. In other cases, when multiple comparisons were considered, One-Way ANOVA was applied. Survival curves of S. littoralis larvae fed with experimental tomato leaf were compared by using Kaplan–Meier and log-rank analysis. The normality of data was checked with the Shapiro–Wilk test and Kolmogorov–Smirnov test, while homoscedasticity was tested with Levene’s test and Barlett’s test. Unpaired Student’s *t*-test or One-Way ANOVA test, followed by Tukey’s post-hoc multiple-comparison test, were used to compare larval weight, respectively, for bioassays with tomato treated plants and tomato ProSys_(1-178)_ transgenic plants. When necessary, the transformation of data was carried out to meet the assumptions of normality and homoscedasticity. When significant effects were observed (*p* < 0.05), Tukey’s post-hoc test was used to compare the mean values. Error bars referring to deviation standards were displayed.

Student’s *t*-test was also used to compare the necrosis area for bioassay with tomato treated plants and tomato ProSys_(1-178)_ transgenic plants. Moreover, the relative quantification of OGs abundance was compared by Student’s *t*-test between experimental samples and controls. Error bars referring to standard error were displayed.

## 3. Results

### 3.1. Production and Characterization of Transgenic Tomato Plants Expressing ProSys_(1-178)_

Tomato plants were stably transformed via *Agrobacterium tumefaciens* using pPRO8 vector [24] in order to gain a construct containing the ProSys cDNA sequence lacking the region coding for the Sys peptide (Figure S1a). A schematic representation of the construct mobilized by *A. tumefaciens* in tomato plants is shown in Figure S1b. Truncated ProSys is under the control of the constitutive CaMV 35SRNA promoter and the pea rbcs terminator (Figure S1b). Putative transformants were screened by PCR (Figure S1c). As expected, genotypes showing different transgene expression levels were obtained (Figure S1d).

Transgenic genotypes homozygous for a single-copy T-DNA insertion and high levels of transgene expression were selected by growing plants up to T_4_ generations on kanamycin-enriched media and analyzing progenies for kanamycin resistance and PCR. Two homozygous lines (indicated as line 1 and line 2) were selected for further investigations. 

**Figure 1:**
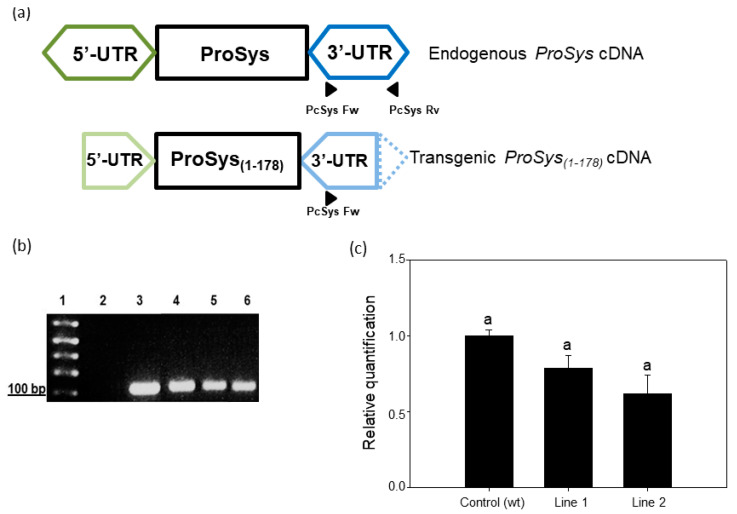
Expression analysis of endogenous ProSys. (**a**) PCR amplification strategy of the endogenous ProSys gene. Primers couple named PcSys Fw and Rv allows the endogenous ProSys to be discriminated from the transgenic expression cassette due to their annealing on the 3′-UTR region (dark blue hexagon); the PcSys reverse primer annealing is impaired on the transgenic cassette due to the truncation of 3′-UTR (dashed part of the light blue pentagon). (**b**) RT-PCR of endogenous ProSys on transgenic and control plants. Lane 1: 1 kb Plus Ladder (Thermo Fisher Scientific); lane 2: control (no template); lane 3: PCR positive control; lane 4: amplification of ProSys cDNA in untransformed plants; lanes 5–6: amplification of ProSys_(1-178)_ lines 1 and 2 with PcSys primers. (**c**) Relative quantification (RQ) of the endogenous Prosystemin gene expression by Real-Time RT-PCR. Amplification has been carried out using primers annealing on 3′-UTR region (truncated in transgenic expression cassette). RQ is shown relative to the calibrator genotype Red Setter. No significant differences in gene expression levels were detected between transgenic and control plants.

In these lines, the amplification of a specific region of the endogenous ProSys gene by Real-Time RT-PCR, using primers annealing on ProSys 3′-UTR (Figure 1a,b, Table S1), ensured that the truncated ProSys did not influence the expression of the endogenous ProSys gene (Figure 1c). Indeed, the quantitative evaluation of the endogenous ProSys gene shown in Figure 1c indicated that the amount of ProSys transcripts is not significantly different among the three samples. This observation can be explained considering that the primer pairs PcSys Fw and Rv exclusively amplify the endogenous ProSys (Figure 1a) that is also present in the transgenic lines. As a matter of fact, these lines, besides the transgenic ProSys transcripts, have also deleted ProSys transcripts; therefore, as expected, they cannot be amplified with the primer pairs PcSys Fw and Rv (Figure S2).

These results indicated the suitability of the transgenic plants to evaluate the effect of the truncated ProSys gene on tomato defense responses.

### 3.2. Production and Characterization of the Biochemical Features of ProSys_(1-178)_

**Figure 2:**
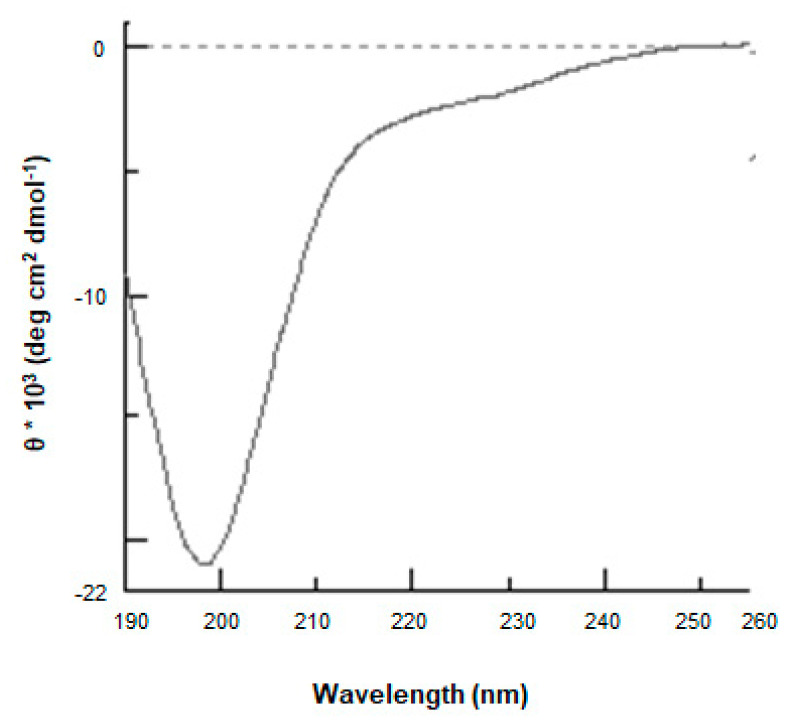
ProSys_(1-178)_ recombinant protein is intrinsically disordered. The Far-UV CD spectrum was recorded from 260 to 190 nm at 20 °C in 10 mM phosphate buffer pH 7.4 at a protein concentration of 6.8 µM.

cDNA encoding ProSys_(1-178)_ was cloned in the pETM11 expression vector designed to obtain a protein having an N-terminal histidine tag. The recombinant protein was expressed in the *E. coli* BL21(DE3) strain and was highly purified after two purification steps, with a final yield of 1 mg/L culture. Protein identity was confirmed by LC-ESI-MS analysis (data not shown). As expected, ProSys_(1-178)_ showed the peculiar features of an IDP, as observed for the full-length protein [20]. In particular, ProSys_(1-178)_ migrated as a protein with a greater molecular weight on SDS-PAGE and eluted as a protein with an apparent molecular mass of 54.4 kDa by SEC (Figure S3a,b). The light-scattering analysis confirmed that, in solution, ProSys_(1-178)_ is a monomer with a molecular weight of 21.1 ± 0.2 kDa (Figure S3c), thus highlighting its scarce compactness as already reported for the full-length protein. Indeed, CD spectra revealed a disorder content (Figure 2), a reversible temperature-induced behavior, and a capability to increase secondary structure content in the presence of TFE co-solvent (Figure S4a,b).

The presence of an isodichroic point at 203.5 was consistent with a coil-helix transition (Figure S4).

### 3.3. ProSys_(1-178)_ Enhances Plant Resistance against S. littoralis and B. cinerea

#### 3.3.1. Transgenic Plants Assays

**Figure 3:**
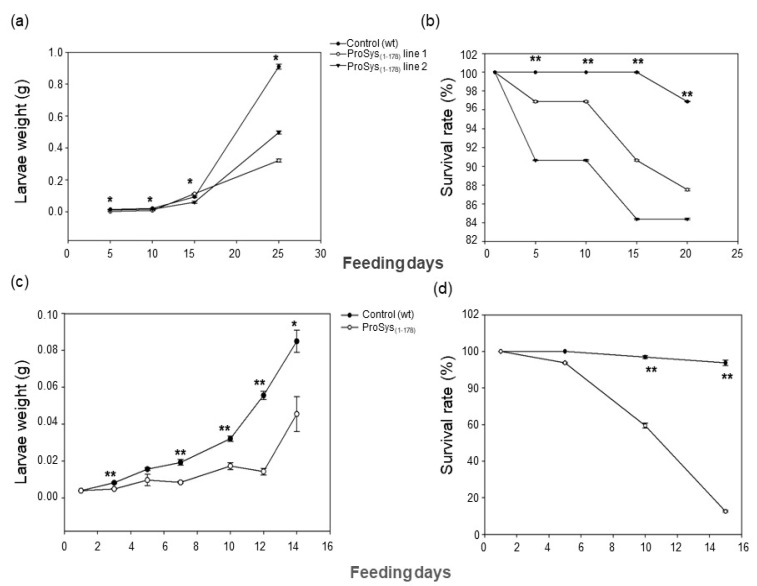
Enhanced resistance of ProSys_(1-178)_ transgenic lines to *S. littoralis* larvae (**a**,**b**) and of leaves of tomato plants exogenously treated with ProSys_(1-178)_ recombinant protein (**c**,**d**). (**a**,**c**) Larval weight increase upon feeding with leaves from ProSys_(1-178)_ transgenic lines (ProSys_(1-178)_ lines 1 and 2) and control plants and upon feeding with leaves from ProSys_(1-178)_-treated and control plants. The graphs display the average (± S.D.) of larval weights on several feeding days (**a**, * One-way ANOVA: *p* < 0.0001; **c**, Student’s *t*-test: * *p* < 0.01; ** *p* < 0.0001). (**b**,**d**) Survival rate of larvae fed on leaves transgenic or control plants and on ProSys_(1-178)_-treated and control leaves (** Log-Rank test: *p* < 0.0001). Asterisks denote mean values that are significantly different.

*S. littoralis* larvae fed with ProSys_(1-178)_ transgenic leaf disks were severely impaired in their growth (Figure 3a) and showed higher mortality rates compared to controls (Log-Rank test: χ^2^ = 21.19, df = 3, *p* < 0.0001) (Figure 3b). From day five until pupation, larval weights were significantly lower for larvae fed on the two transgenic lines than on controls (Figure 3a, Table S2) (One Way ANOVA: *p* < 0.0001). 

**Figure 4:**
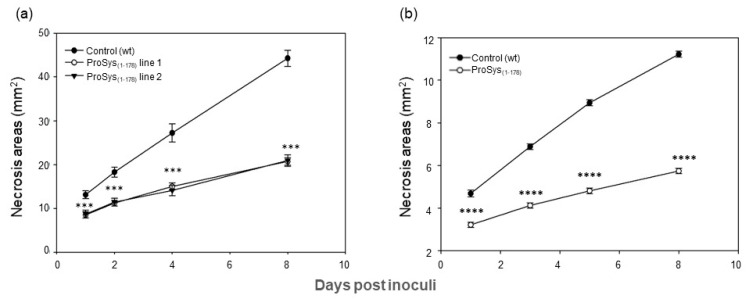
Enhanced resistance of ProSys_(1-178)_ transgenic lines and in ProSys_(1-178)_-treated tomato leaves to *B. cinerea*. (**a**) Dimension of necrosis areas in leaves of control plants and of ProSys_(1-178)_ transgenic lines 1 and 2. (**b**) Response to *B. cinerea* infection in leaves from mock-treated and ProSys_(1-178)_ treated plants. The graphs display the average (±S.E.) of the lesion size at several days post inoculum (hpi). Statistical analysis was performed by Student’s *t*-test (*** *p* < 0.001; **** *p* < 0.0001). Asterisks denote mean values that are significantly different.

The transgene effect also caused a strong reduction of *B. cinerea* colonization with the consequent reduction of necrosis areas (Figure 4a).

#### 3.3.2. Plant Treatments with Exogenous ProSys_(1-178)_

The exogenous application of recombinant protein ProSys_(1-178)_ confirmed a direct role of the protein in triggering plant defense responses. Indeed, *S. littoralis* larvae fed with ProSys_(1-178)_-treated leaves showed, compared to controls, a significant reduction of their weight starting from day 3 until day 14 (Student’s *t*-test: *p* < 0.0001) (Figure 3c, Table S3), and a higher mortality rate, which reached 100% by day 15 (Log-Rank test: χ^2^ = 59.75, df = 1, *p* < 0.0001) (Figure 3d). Similarly, the effect of the plant treatment with the recombinant ProSys_(1-178)_ on *B. cinerea* resulted in a significant reduction in lesion areas (Figure 4b).

### 3.4. The Transcriptomic Profiles of Tomato Plants Is Strongly Influenced by ProSys_(1-178)_ Expression

The transcriptomic changes imposed by the constitutive overexpression of the truncated ProSys were monitored by using the Tomato Gene Expression 4 × 44 K array (Agilent Technologies). A comparative gene expression analysis was performed with cDNAs from leaves of the two selected transgenic genotypes (ProSys_(1-178)_ lines 1 and 2) and Red Setter untransformed controls. The expression of the ProSys_(1-178)_ in tomato imposed a strong modification of the transcriptomic profile, up-regulating 428 and down-regulating 537 transcripts. The classification of the differentially expressed genes (DEGs), based on the ontological domain “biological process”, is shown in Figure S5. Several defense-related functional categories, such as “response to abiotic stimulus”, “response to biotic stimulus”, “secondary metabolic process” and “cell death” were modified in transgenic plants. Tables S4 and S5 list all differentially expressed transcripts identified in ProSys_(1-178)_ plants. The identification of pathways including DEGs was carried out using KEGG analysis (Table S6). Defense-related pathways affected by ProSys_(1-178)_ expression were those involved in flavonoid biosynthesis and glutathione metabolism (Table S6). 

Microarray data were validated by monitoring the expression of a group of genes by Real-Time RT-PCR, confirming what was observed through the array (Figure S6).

Tomato genes whose transcripts were modulated by the constitutive expression of truncated ProSys were grouped according to their functional annotation (Tables S4 and S5).

#### 3.4.1. Defense-Related Genes

A wide array of genes involved in early signaling responses were up-regulated, such as four genes associated with the oxidative burst (i.e., glutathione-S-transferase (GST), NADH dehydrogenase, laccase 22 and metacaspase 7), two transcripts of one gene coding for polygalacturonase (PG) and a long list of transcripts encoding for kinases, phosphatases (including a dual-specificity phosphatase 1, DUSP1) and calcium-related proteins (Table 1). Other transcripts encoding for proteins involved in the early stages of defense responses were down-regulated, including members of the GST family, peroxidases, catalases and calmodulin (Table 1 and Table S5). A wide group of DEGs are associated with responses to abiotic stresses, such as high temperature, and include transcripts coding several types of chaperone proteins, heat shock protein 4 and 70, stress-related protein (SRP), DNAj heat shock proteins and dehydration-responsive family protein (Table 1).

#### 3.4.2. Anatomical Defensive Structure

ProSys_(1-178)_ plants showed the up-regulation of genes involved in anatomical defensive structure, such as those associated with the strengthening of physical barriers, such as callose, cellulose synthases, and hydroxyproline-rich glycoprotein family, which participate in the formation of cell wall appositions designed to prevent or retard pathogen infiltration (Table 2) [32]. In addition, the strong up-regulation of transcripts encoding a PG enzyme (two transcripts related to the same gene) and immune-responsive cytoskeletal elements, such as kinesin, actin, villin 2 and villin 4, further indicated the promotion of processes leading to cell wall re-organization (Table 2).

#### 3.4.3. Secondary Metabolism

Plant antibiosis and antixenosis are often entrusted to secondary metabolism, which was altered in ProSys_(1-178)_ plants (Figure S5). The most representative secondary metabolism-related DEGs were found to be involved in flavonoid biosynthesis (i.e., crocetin, dihydroflavonol 4-reductase, chalcone synthase, flavanone 3 beta-hydroxylase) (Table 3). Another remarkable group of DEGs was involved in phenylpropanoid biosynthetic pathways. Interestingly, many phenylpropanoids show anti-microbial and anti-fungal activities [33,34]. In addition, the stress-related polyamines family was also affected, as shown by the up-regulation of the transcript of a putrescine-interacting protein and the down-regulation of an ornithine decarboxylase gene, respectively (Table 3).

#### 3.4.4. Hormone-Related Pathways

Remarkably, numerous enzymes of the biosynthetic pathway of the three major plant hormones involved in defense responses, such as JA, salicylic acid (SA) and ethylene (ET), were down-regulated (Tables S4 and S5). For example, transcripts involved in JA biosynthesis (i.e., 13-lipoxygenase and other classes of lipases), as well as members of the JA-responsive gene family (i.e., wound-inducible, Kunitz trypsin inhibitor, proteinase inhibitor I and metallocarboxypeptidase inhibitors) were down-regulated. Similarly, transcripts involved in SA methylation (i.e., S-adenosylmethionine-dependent methyltransferase) and SA-responsive genes (chitinase, osmotin, subtilisin and PR1a and PR1b, the two latter may also be responsive to ET) were also down-regulated (Table 4) [35,36,37]. A gene involved in ET biosynthesis, coding for 1-aminocyclopropane-1-carboxylate oxidase, was also down-regulated, whereas other ET-related transcripts coding for a serine/ threonine (Ser/Thr) kinase and an ethylene receptor were up-regulated (Table 4). Nine genes associated with the auxin pathway were down-regulated. An analogous trend was observed for genes involved in the gibberellin pathway, as shown by the down-regulation of six transcripts involved in their biosynthesis and perception (Table 4).

### 3.5. Defense-Related Genes Are Up-Regulated in ProSys_(1-178)_-Treated Plants

**Figure 5:**
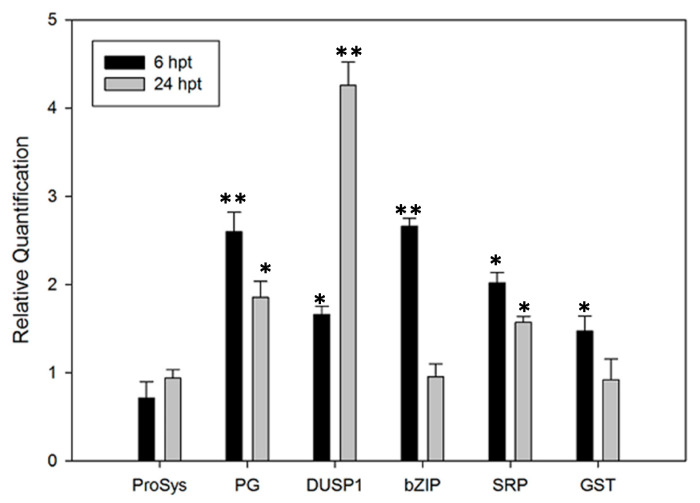
Relative expression of defense-related genes in tomato plants treated with exogenous ProSys_(1-178)_ recombinant protein. Relative quantification of several defense-related genes induced at 6 and 24 h after plant treatment (hpt) with 100 pM ProSys_(1-178)_. The genes under investigation were: *ProSystemin, ProSys; Polygalacturonase, PG; dual-specificity phosphatase 1, DUSP1; basic leucine zipper protein family, bZIP; stress-related protein, SRP; glutathione S-transferase, GST.* Quantities are relative to the calibrator represented by mock-treated plants. Statistical analysis was performed by Student’s *t*-test (* *p* < 0.05. ** *p* < 0.01). Asterisks denote mean values that are significantly different.

The effect of the ProSys_(1-178)_ exogenous supply on tomato plants was evaluated by monitoring the expression of five defense-related genes, selected among the genes up-regulated in transgenic plants and listed in Table S4, coding for polygalacturonase (Solyc08g082170.2.1), dual-specificity phosphatase 1 (Solyc05g054700.2.1), basic leucine zipper protein family (Solyc01g090270.2.1), stress-related protein (Solyc09g074930.2.1) and glutathione S-transferase (Solyc09g011500.2.1). We included in this gene set *ProSys* (Solyc05g051750.2.1) to show it in comparison with the other genes. Transcript accumulation was analyzed 6 and 24 h after ProSys_(1-178)_ application. Notably, as shown in Figure 5, all genes, except ProSys, were significantly over-expressed.

### 3.6. ProSys_(1-178)_ Induces the Release of Oligogalacturonides

**Figure 6:**
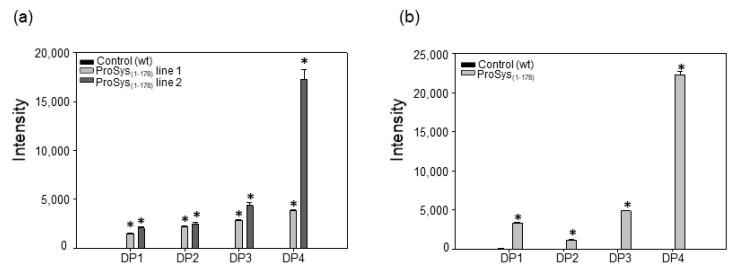
OGs identified in transgenic ProSys_(1-178)_ plants (**a**) and in ProSys_(1-178)_-treated plants (**b**).The graphs display the means of different DP (± S.D.) of three biological replicates. DP = degree of polymerization. Statistical analysis was performed by Student’s *t*-test (* *p* < 0.001).

In order to verify if the overexpression of PG observed both in transgenic ProSys_(1-178)_ and in ProSys_(1-178)_-treated plants were associated with the release of OGs; we analyzed the leaves for OG presence by MALDI-TOF analysis. Figure S7 shows an example of MALDI-TOF spectra of the transgenic lines ProSys_(1-178)_ line 2 (black) and relative control (green). We found signals attributable to OGs up to four for the degree of polymerization (DP) in transgenic plant samples, and their relative quantification was obtained by comparing the peak intensity for each signal in the spectrum (Figure 6a). In all samples, the OGs with four DP were the most abundant, especially in both ProSys_(1-178)_ lines. Similarly, in all ProSys_(1-178)_-treated samples, after 6 h from the treatment, we found signals attributable to OGs with four DP (Figure 6b). Interestingly, both transgenic and treated plants released OGs of a similar length.

## 4. Discussion

Plants have evolved different families of functionally related peptide signals involved in defense responses against insects and pathogens. These peptides are often released by larger precursors and are perceived by membrane receptors, which activate defense signaling cascades [38,39]. One of the best-characterized signaling peptides is tomato Sys, which is released from ProSys upon wounding and herbivory. Sys interacts with a leucine-rich repeat receptor kinase [40] to trigger wound and defense responses in tomato [1,2,3,10,11,12,14,16,17]. 

Despite these observations, recent findings describing the disordered structure of ProSys [20] suggested that, besides Sys, other ProSys regions could have roles in defense responses. Intrinsically disordered sequences allow proteins to bind multiple molecular partners with often different functional outcomes [41,42]. This mechanism may explain the ability of ProSys to protect plants against a wide array of stresses [42]. This reasoning, and previous work, showing proteomic reprogramming and enhanced resistance against fungi by tobacco plants expressing ProSys_(1-178)_ [24], stimulated the hypothesis that ProSys is more than a simple precursor. Here we demonstrate that *S. littoralis* larvae fed on ProSys_(1-178)_ leaves had reduced growth and vitality and that *B. cinerea* infection is strongly limited on ProSys_(1-178)_ plants. Interestingly, these results are confirmed by plant treatments with the recombinant ProSys_(1-178)_ protein. These findings are in agreement with the consistent transcriptomic reprogramming observed in transgenic plants constitutively expressing the gene devoid of Sys coding sequence and with the expression profiles of defense-related genes evidenced in plants treated with recombinant ProSys_(1-178)_. As expected for the constitutive expression of a sequence associated with the plant immune system, metabolic and cellular processes were largely affected. The multilevel distribution of GO categories indicated a cellular reprogramming involving primary and secondary metabolism, with a clear influence on defense mechanisms that included the up-regulation of genes activated in the early defense, such as GST (Table 1), associated with the regulation of the oxidative burst. Plant glutathione S-transferases are ubiquitous multifunctional enzymes encoded by large gene families that participate in ROS scavenging, stress tolerance, detoxification of toxic substances, plant growth and development, both in vivo and in vitro [43,44,45,46]. Their fulfillment of such an ample number of functions might explain the observed up and down-regulation of different members of this gene family in the transgenic plants. Several other genes associated with the initial stages of the defense signaling cascade were up-regulated, such as kinases, phosphatases and calcium-related proteins. These genes are major milestones of defense response activation [47,48]. For example, the expression of *DUSP1* (Table 1) is induced by cellular stresses and modulates selected MAP kinases, which are important plant defense components [49]. Protein kinases and Ca^2+^ binding proteins play important roles in mediating defense responses against herbivores, while receptor-like kinases play a central role in pathogen recognition and the subsequent activation of plant defense mechanisms [50,51]. Other increased transcripts code for members of the hydroxyproline-rich glycoprotein family (HRGPs) (Table 2), a superfamily of cell wall proteins, involved in stress responses, signaling and molecular recognition pathways [52]. Intriguingly, tomato HRGP (LeproHypSys), the protein precursor of HypSys I, II and III peptides, which is up-regulated by wounding, Sys and methyl jasmonate treatments [53], and associated with Sys in the coordination of tomato defense responses [9], has characteristic features of IDPs, similarly to ProSys [52]. Thus, these two IDPs may promote the establishment of a defense protein network active in protecting tomato plants against a wide array of biotic stressors. Interestingly, HRGPs appear to participate in cell wall appositions, to prevent or retard pathogen infiltration [32]. Indeed transcriptomic results suggest that ProSys_(1-178)_ highly potentiates tomato physical barriers up-regulating a conspicuous group of genes related to cell-wall reinforcement and callose synthesis (Table 2). It was previously demonstrated that the constitutive expression of callose synthase confers SA and JA-independent resistance to powdery mildew in Arabidopsis [54]. Similarly, ProSys_(1-178)_ plants, in which SA and JA defense pathways are down-regulated, showed the up-regulation of Callose Synthase 11 (Table 2). According to these data the improved tolerance to *B. cinerea* shown by ProSys_(1-178)_ plants could be associated with the strengthening of physical barriers, independently from hormone-regulated pathways. In this context, the abundance of transcripts coding for a PG enzyme likely led to the production of OGs, as observed both in transgenic and in treated leaves, which contributed to the observed resistance of ProSys_(1-178)_ plants against *B. cinerea*, as recently observed in Arabidopsis [55]. The crucial role of the cytoskeleton and its re-organization during plant–pathogen interactions has been widely reported [56,57,58,59]. Thus, the up-regulation of genes coding for kinesin actin, and villin 2 and villin 4 observed in ProSys_(1-178)_ plants suggests an extensive cytoskeleton re-organization (Table 2). Especially in the defense against fungi and oomycetes, actin dynamics represents one of the key components in the formation of the physical barriers against their penetration [60]. In addition to the reinforcement of physical barriers, the resistance of ProSys_(1-178)_ plants against insect pests could be the result of the modifications affecting secondary metabolism (Table 3). The KEGG analysis helped to address four DEGs to the flavonoid biosynthesis pathway. These compounds are known to defend plants against various biotic and abiotic stresses, including UV radiation, pathogens and insect pests [61]. Among them is crocetin, an isoprenoid precursor of terpenoids, the largest group of plant chemicals with a primary role in plant growth and development, but also active in counteracting abiotic and biotic stressors [62]. Moreover, this molecule has been recently associated with plant–virus interactions [63]. Taken together, these observations explain the increased tolerance of the plants expressing ProSys_(1-178)_ protein towards *B. cinerea* and *S. littoralis*, since cell-wall reinforcement and phenylpropanoid-derived compounds are well recognized for their anti-feedant and cytotoxic effects on insect pests and pathogens [64].

Notably, transgenic plants showed the suppression of the major hormone-regulated pathways related to plant defense responses (i.e., JA, SA and ET), as indicated by the down-regulation of genes coding for lipoxygenase (LOX), PIs, pathogenesis-related proteins (PRs) and 1-aminocyclopropane-1-carboxylate oxidase (ACCO), a key enzyme involved in ET biosynthesis (Table 4).

It is worth noting that ET perception occurs through hormones binding to receptors, which act as negative regulators of ethylene response. When the hormone is absent, ET receptors activate proteins of the serine/threonine kinase family that act by phosphorylating a specific protein in order to repress its ability to induce ethylene responses [65]. In our results, genes coding for both (Ser/Thr) kinase and an ethylene receptor were up-regulated coherently with the down-regulation of ACCO (Table 4). In addition, genes involved in auxin and gibberellin pathways were also down-regulated (Table 4). Considering that the Sys signal transduction pathway, which leads to the production of JA, is activated by the interaction of Sys with its receptor [40], the lack of up-regulation of JA-dependent genes in ProSys_(1-178)_ plants was not surprising since Sys was missing in the deleted genes that did not activate the endogenous *ProSys*. This gene is the major actor of the activation of genes involved in multiple hormone-signaling pathways associated with plant defense [12,14,15,24]. However, why the hormone-related defense pathways are mainly down-regulated in ProSys_(1-178)_ plants need further clarification. As mentioned, a remarkable up-regulation of PG-related transcripts was observed in transgenic plants. These enzymes depolymerase pectin, one of the major plant cell wall components producing pectin-derived OGs. OGs are perceived by cell wall-associated kinases and activate the plant’s innate immunity [66,67]. The down-regulation of the auxin biosynthetic pathways observed in ProSys_(1-178)_ plants is consistent with previous observations reporting that OGs have an antagonistic role to auxin [68,69]. This antagonism may play an important role in prioritizing defense vs. growth, thus allowing plants to allocate energy to the defense mechanisms if required. Interestingly, PG was up-regulated in plants treated with ProSys_(1-178)_ recombinant protein, confirming that transcripts of this family are selectively up-regulated by ProSys_(1-178)_ protein since their expression profiles were not altered in the transcriptome of transgenic plants expressing Prosystemin as analyzed through microarrays [12]. Therefore, it appears that ProSys_(1-178)_ is not only biologically active but that it operates via a mechanism that is able to activate defense pathways that involve OGs. OGs are key components of DAMP signaling able to elicit, in several plant species, a wide range of defense responses [55,66]. They are thought to be released from plant cell walls upon partial degradation of homogalacturonan, originating during microbial infections, by microbial PGs [70] or by the action of endogenous PGs induced by mechanical damage [71]. Very recently, it was shown that OGs are also produced by the activity of pectin lyases, at least in Arabidopsis [55].

One structural requirement for the biological activity of OGs is the degree of polymerization. Although it has been suggested that long OGs, with a degree of polymerization (DP) between 10 and 20 (DP > 10) are the most effective in modulating plant defense signaling responses [72,73,74], it was shown that short OGs (DP < 10) influence plant defense [75]. For example, short OGs (DP4-6, DP2 and DP1-7, respectively) induced genes involved in pathogen response in tomato and potato [76,77,78,79,80]. Therefore, we propose that the short OGs produced both in ProSys_(1-178)_ transgenic and treated plants actively contribute to defense responses.

Moreover, the up-regulation in ProSys_(1-178)_-treated plants of a member of bZIP transcription factor (TF) (Solyc01g090270.2.1) family, 6 h post plant treatment confirming the early involvement of bZIP TF in plant defense responses, in line with the key-roles played by TF in plant innate immunity [81,82], was remarkable.

## 5. Conclusions

Collectively, the experimental evidence gathered so far clearly demonstrates that ProSys_(1-178)_ proteins trigger tomato plant defense responses by activating specific classes of defense-related genes and prioritizing defense with respect to growth. It is tempting to speculate that the overall function of the wild-type ProSys is to activate at least two tomato defense pathways, the Sys-dependent pathway connected with the induction of JA biosynthesis and the successive activation of a set of defense-related genes, and the ProSys_(1-178)_-dependent pathway associated with OGs production, leading to the OGs mediating the plant’s immunity. Further studies are required to confirm this hypothesis. This presumed mechanism may explain the large “anti-stress” capacity of ProSys. 

## Figures and Tables

**Table 1:** Examples of defense-related genes up and down-regulated in ProSys_(1-178)_ lines.

Gene ID	logFC	Description
Solyc09g011630.2.1	2,097,059	Glutathione S-transferase
Solyc09g011500.2.1	36,160,662	Glutathione S-transferase
Solyc02g092270.2.1	24,208,286	NADH dehydrogenase
Solyc03g083900.2.1	23,965,964	Laccase-22
Solyc09g098150.2.1	2,361,944	Metacaspase 7
Solyc08g082170.2.1	6,065,851	Polygalacturonase
Solyc05g054700.2.1	31,435,359	Dual-specificity phosphatase 1
Solyc01g081250.2.1	−43,141,214	Glutathione-S-transferase
Solyc01g104860.2.1	−5,063,558	Peroxidase 43
Solyc01g105070.2.1	−3,189,162	Peroxidase
Solyc07g017880.2.1	−23,453,817	Peroxidase
Solyc09g007270.2.1	−2,231,321	Ascorbate peroxidase
Solyc12g094620.1.1	−27,919,602	Catalase
Solyc03g115930.1.1	−221,963	Calmodulin-like protein
Solyc01g010020.2.1	−21,430,988	Calmodulin
Solyc01g103450.2.1	22,321,863	Chaperone DnaK
Solyc07g006540.2.1	21,124,113	Chaperone protein ClpB
Solyc04g081570.2.1	39,538,085	Chaperone protein htpG
Solyc11g065260.1.1	25,225,124	Chaperone protein dnaJ
Solyc02g080470.2.1	2,050,839	Heat shock protein 4
Solyc12g043110.1.1	23,375	Heat shock protein 70
Solyc09g074930.2.1	52,265,615	Stress-related protein
Solyc04g081070.2.1	21,339,486	Heat shock protein DnaJ domain protein
Solyc04g063230.2.1	20,030,978	Dehydration-responsive family protein
Solyc06g069870.2.1	22,527,552	Dehydration-responsive family protein
Solyc03g005600.2.1	23,916,254	Dehydration-responsive protein

Values of Log2 Fold Change (logFC) and gene description are indicated.

**Table 2:** Examples of genes involved in anatomical defensive structure in ProSys_(1-178)_ lines.

Gene ID	logFC	Description
Solyc02g078230.1.1	25,605,323	Callose synthase 11
Solyc12g056580.1.1	23,134,887	Cellulose synthase
Solyc04g071650.2.1	22,216,854	Cellulose synthase
Solyc03g097050.2.1	2,168,395	Cellulose synthase
Solyc08g061100.2.1	20,450,845	Cellulose synthase
Solyc01g087210.2.1	2,039,101	Cellulose synthase
Solyc05g009930.2.1	2,154,703	Hydroxyproline-rich glycoprotein family protein
Solyc08g082170.2.1	6,065,851	Polygalacturonase
Solyc02g084390.2.1	2,097,367	Kinesin protein nack1
Solyc06g009780.2.1	22,364,771	Kinesin
Solyc11g005330.1.1	20,083,592	Actin
Solyc04g015830.2.1	20,667,365	Villin 2
Solyc02g021420.2.1	28,023,307	Villin-4

Values of Log2 Fold Change (logFC) and gene description are indicated.

**Table 3:** Examples of genes involved in secondary metabolism in ProSys_(1-178)_ lines.

Gene ID	logFC	Description
Solyc12g098590.1.1	37,859,063	Crocetin chloroplastic-like
Solyc02g085020.2.1	36,648,946	Dihydroflavonol 4-reductase
Solyc05g053550.2.1	25,119,457	Chalcone synthase
Solyc09g091510.2.1	21,222,255	Chalcone synthase
Solyc02g083860.2.1	2,006,604	Flavanone 3 beta-hydroxylase
Solyc08g005860.2.1	22,907,643	Putrescine-binding periplasmic protein
Solyc04g082030.1.1	−2,169,399	Ornithine decarboxylase

Values of Log2 Fold Change (logFC) and gene description are indicated.

**Table 4:** Examples of hormone-related pathway genes up and down-regulated in the ProSys_(1-178)_ lines.

Gene ID	logFC	Description
Solyc01g099210.2.1	−20,045,187	Lipoxygenase
Solyc01g111960.2.1	−52,605,577	GDSL esterase/lipase
Solyc05g043320.1.1	−22,984,564	GDSL esterase/lipase
Solyc11g051060.1.1	−2,308,117	GDSL esterase/lipase 2
Solyc02g090940.2.1	−20,605,335	Lipase
Solyc03g093360.2.1	−21,316,884	Wound/stress protein
Solyc03g098740.1.1	−24,175,534	Kunitz trypsin inhibitor
Solyc09g084470.2.1	−62,815,356	Proteinase inhibitor I
Solyc07g007250.2.1	−14,125,946	Metallocarboxypeptidase inhibitor
Solyc07g007260.2.1	−25,874,014	Metallocarboxypeptidase inhibitor
Solyc04g040180.2.1	−32,112,498	S-adenosylmethionine-dependent methyltransferase
Solyc01g097270.2.1	−23,048,432	Chitinase
Solyc08g080650.1.1	−30,336,623	Osmotin
Solyc06g065370.2.1	−2,287,122	Subtilisin
Solyc09g006010.2.1	−39,370,556	Pathogenesis related protein PR-1
Solyc00g174340.1.1	−31,596,904	Pathogenesis-related protein 1b
Solyc09g089580.2.1	−2,396,617	1-aminocyclopropane-1-carboxylate oxidase
Solyc01g059860.2.1	21,835,773	Serine threonine-protein kinase
Solyc11g006180.1.1	24,757,214	Ethylene receptor
Solyc01g110800.2.1	−33,291,337	Auxin-induced SAUR-like protein
Solyc01g110940.2.1	−21,495,044	Auxin-induced SAUR-like protein
Solyc02g077880.2.1	−32,065,403	Auxin-repressed protein
Solyc02g082450.2.1	−2,151,276	Auxin efflux carrier family protein
Solyc04g082830.2.1	−2,753,833	Auxin efflux carrier family protein
Solyc03g082510.1.1	−2,208,86	Auxin-responsive family protein
Solyc05g008850.2.1	−4,863,605	Auxin responsive protein
Solyc10g052530.1.1	−24,981,644	Auxin-responsive protein
Solyc06g053260.1.1	−47,414,575	Auxin-responsive family protein
Solyc05g051660.1.1	−2,158,873	Gibberellin receptor GID1L2
Solyc09g075670.1.1	−2,292,731	Gibberellin receptor GID1L2
Solyc06g007890.2.1	−2,320,646	Gibberellin-regulated protein
Solyc11g017440.1.1	−55,233,316	Gibberellin-regulated protein 9
Solyc11g011210.1.1	−34,478,865	Gibberellin regulated protein
Solyc07g056670.2.1	−20,980,172	Gibberellin 2-oxidase 2

Values of Log2 Fold Change (logFC) and gene description are indicated.

## Data Availability

The data presented in this study are available in this article and Supplementary Material. Microarray data are available in the Gene Expression Omnibus (GEO) repository under the accession number GSE193362.

## Supplementary Materials

The following are available online at https://www.mdpi.com/article/10.3390/biology11010124/s1, Figure S1. Molecular analysis of ProSys_(1-178)_ transgenic plants. (a) ProSys full-length coding sequence. Red letters highlight Sys coding sequence, and the bold sequence represents the deleted part in ProSys_(1-178)_ cDNA. (b) Schematic representation of the pPRO8 T-DNA binary vector used for plant genetic transformation. LB: T-DNA left border sequence; 35S: CaMV35S RNA gene promoter; ProSys_(1-178)_: Prosystemin cDNA sequence lacking the Sys coding region; rbcS: terminator of the pea rubisco small subunit; nptII: neomycin phosphotransferase II coding sequence; nos: nopaline synthase promoter; RB: T-DNA right border sequence. Black triangles indicate the location of the primers P1 (Sys Fw) and P2 (Sys Rv) used for the screening of transgenic plants. Empty triangles indicate the location of the primers P3 (Sysdel Fw) and P4 (Sysdel Rv) used for the relative quantification of ProSys_(1-178)_ expression. (c) PCR screening of transgenic plants. Lane 1: 1 kb Plus Ladder (Thermo Fisher Scientific); numbers on the left margin indicate the expected marker fragment size. In base pairs; lane 2: control (no template); lane 3: pPRO8 binary vector; lanes 4–10: amplification products from DNA isolated from putative transgenic plants. (d) Relative quantification (RQ) of ProSys_(1-178)_ expression (by real-time RT-PCR). Numbers indicate the genotype corresponding to single transformation events. Primers annealing on ProSys N-terminal region (Sysdel Fw and Sysdel Rv) allow the amplification on both transgenic and untransformed plants. RQ is relative to the calibrator genotype represented by the cDNA from the tomato “Red Setter” cultivar. Figure S2. Schematic representation of PcSys Fw and Rv annealing positions on ProSys 3′-UTR sequence. PcSys reverse primer annealing is impaired on transgenic ProSys transcripts due to the truncation of the 3′-UTR region (italic). Figure S3. Features of purified recombinant ProSys_(1-178)_. (a) 15% SDS-PAGE visualized by Coomassie Brilliant Blue. M: molecular mass marker (20–250 kDa); 1: purified ProSys_(1-178)_. (b) SEC calibration curve built using five standard proteins (red crosses); data for ProSys_(1-178)_ (full circle) were calculated from this curve; (c) SEC-MALS-QELS of ProSys_(1-178)_ confirmed that the protein is a monomer in solution. Figure S4. Structural changes in ProSys_(1-178)_ analyzed by far-UV CD. (a) CD spectra at different temperatures (10, 20, 30, 40, 5 and 60 °C) recorded from 260 to 190 nm at 20 °C in 10 mM phosphate buffer, pH 7.4. The ellipticity at 222 nm vs. temperature is shown in the inset. (b) CD spectra in the presence of increasing TFE concentration (0%, 15%, 20%, 25%, 30% and 35%) recorded from 260 to 190 nm at 20 °C. The ellipticity at 222 nm vs. TFE percentage is shown in the inset. Figure S5. Multilevel distribution of GO categories associated with DEGs identified in ProSys_(1-178)_ plants. GO terms associated with up-regulated (red bars), and down-regulated (green bars) genes are based on the “Biological Process” ontological domain. Figure S6. Microarray validation by Real-Time RT-PCR. Relative quantification of DEGs identified through microarray analysis. Genes under investigation were: *3-oxo-5-alpha-steroid 4-dehydrogenase family protein, PPR2; MLO-like protein 3, MLO3; Crocetin UDP-glucosyltransferase family 1 protein, crocetin; stress-related protein, SRP; dual-specificity phosphatase, DUSP1; 1 leucine aminopeptidase 1A. Lap1A; mate efflux carrier protein, MATE*. Data were calibrated on the “Red Setter” untransformed genotype. Statistical analysis was performed by Student’s *t*-test (* *p* < 0.05; ** *p* < 0.01). Figure S7. OGs identified in ProSys_(1-178)_ plants. MALDI-TOF spectra of the transgenic lines ProSys_(1-178)_ line 2 (black) and its relative control RS1 (green). DP = degree of polymerization. Table S1. Primers and their main features. Table S2. Weight increase of *S. littoralis* larvae fed on leaves of ProSys_(1-178)_ transgenic plants. The leaves employed were taken from control plants (C) and from two transgenic plant lines: ProSys_(1-178)_ lines 1 and 2, respectively. Average values ± standard deviations are reported for each sample for all experimental time points analyzed. For each time point, mean values denoted with different letters are significantly different (* One-way ANOVA: *p* < 0.0001 followed by Tukey’s post-hoc multiple-comparison test). Table S3. Weight increase of *S. littoralis* larvae fed on leaves of tomato plants treated with ProSys_(1-178)_ recombinant protein. Averages ± standard deviations are reported for ProSys_(1-178)_-treated and control (C) samples for all experimental time points. (Student’s *t*-test: * *p* < 0.01; ** *p* < 0.0001). Table S4. Differentially expressed genes (DEGs) of ProSys_(1-178)_ transgenic plants identified by microarray analysis (Tomato array 4 × 44 K, Agilent Technologies). Probe Name, Locus ITAG2.5, Fold change, Best Blast Hit Descriptor for up-regulated genes are reported. Table S5. Differentially expressed genes (DEGs) of ProSys_(1-178)_ transgenic plants identified by microarray analysis (Tomato array 4 × 44 K, Agilent Technologies). Probe Name, Locus ITAG2.5, Fold change, Best Blast Hit Descriptor for down-regulated genes are reported. Table S6. KEGG analysis of the differentially expressed genes. Mapping of enzymatic activities (Enzyme and Enzyme ID) of the DEGs (Sequence ID) in the KEGG pathways (Pathway).

## References

[1] McGurl B., Orozco-Cardenas M., Pearce G., Ryan C.A. (1994). Overexpression of the prosystemin gene in transgenic tomato plants generates a systemic signal that constitutively induces proteinase inhibitor synthesis. Proc. Natl. Acad. Sci. USA.

[2] Pearce G., Strydom D., Johnson S., Ryan C.A. (1991). A polypeptide from tomato leaves induces wound-inducible proteinase inhibitor proteins. Science.

[3] Ryan C.A. (2000). The systemin signaling pathway: Differential activation of plant defensive genes. Biochim. Biophys. Acta Protein Struct. Mol. Enzymol..

[4] Beloshistov R.E., Dreizler K., Galiullina R.A., Tuzhikov A.I., Serebryakova M.V., Reichardt S., Shaw J., Taliansky M.E., Pfannstiel J., Chichkova N.V. (2018). Phytaspase-mediated precursor processing and maturation of the wound hormone systemin. New Phytol..

[5] McGurl B., Pearce G., Orozco-Cardenas M., Ryan C.A. (1992). Structure, expression, and antisense inhibition of the systemin precursor gene. Science.

[6] Schilmiller A.L., Howe G.A. (2005). Systemic signaling in the wound response. Curr. Plant Biol..

[7] Pearce G., Bhattacharya R., Chen Y.-C. (2008). Peptide signals for plant defense display a more universal role. Plant Signal. Behav..

[8] Pearce G. (2011). Systemin, hydroxyproline-rich systemin and the induction of protease inhibitors. Curr. Protein Pep. Sci..

[9] Narváez-Vásquez J., Orozco-Cárdenas M.L., Ryan C.A. (2007). Systemic wound signaling in tomato leaves is cooperatively regulated by systemin and hydroxyproline-rich glycopeptide signals. Plant Mol. Biol..

[10] Corrado G., Sasso R., Pasquariello M., Iodice L., Carretta A., Cascone P., Ariati L., Digilio M., Guerrieri E., Rao R. (2007). Systemin regulates both systemic and volatile signaling in tomato plants. J. Chem. Ecol..

[11] Degenhardt D.C., Refi-Hind S., Stratmann J.W., Lincoln D.E. (2010). Systemin and jasmonic acid regulate constitutive and herbivore-induced systemic volatile emissions in tomato, Solanum lycopersicum. Phytochemistry.

[12] Coppola M., Corrado G., Coppola V., Cascone P., Martinelli R., Digilio M.C., Pennacchio F., Rao R. (2015). Prosystemin overexpression in tomato enhances resistance to different biotic stresses by activating genes of multiple signaling pathways. Plant Mol. Biol. Rep..

[13] Dombrowski J.E. (2003). Salt stress activation of wound-related genes in tomato plants. Plant Physiol..

[14] Zhang H., Zhang H., Lin J. (2020). Systemin-mediated long-distance systemic defense responses. New Phytol..

[15] Orsini F., Cascone P., De Pascale S., Barbieri G., Corrado G., Rao R., Maggio A. (2010). Systemin-dependent salinity tolerance in tomato: Evidence of specific convergence of abiotic and biotic stress responses. Physiol. Plant.

[16] El Oirdi M., El Rahman T.A., Rigano L., El Hadrami A., Rodriguez M.C., Daayf F., Vojnov A., Bouarab K. (2011). *Botrytis cinerea* manipulates the antagonistic effects between immune pathways to promote disease development in tomato. Plant Cell.

[17] Coppola M., Lelio I.D., Romanelli A., Gualtieri L., Molisso D., Ruocco M., Avitabile C., Natale R., Cascone P., Guerrieri E. (2019). Tomato plants treated with systemin peptide show enhanced levels of direct and indirect defense associated with increased expression of defense-related genes. Plants.

[18] Dombrowski J.E., Pearce G., Ryan C.A. (1999). Proteinase inhibitor-inducing activity of the prohormone prosystemin resides exclusively in the C-terminal systemin domain. Proc. Natl. Acad. Sci. USA.

[19] Ryan C.A., Pearce G. (2003). Systemins: A functionally defined family of peptide signals that regulate defensive genes in Solanaceae species. Proc. Natl. Acad. Sci. USA.

[20] Buonanno M., Coppola M., Di Lelio I., Molisso D., Leone M., Pennacchio F., Langella E., Rao R., Monti S.M. (2018). Prosystemin, a prohormone that modulates plant defense barriers, is an intrinsically disordered protein. Protein. Sci..

[21] Oldfield C.J., Meng J., Yang J.Y., Yang M.Q., Uversky V.N., Dunker A.K. (2008). Flexible nets: Disorder and induced fit in the associations of p53 and 14-3-3 with their partners. BMC Genom..

[22] Hamdi K., Salladini E., O’Brien D.P., Brier S., Chenal A., Yacoubi I., Longhi S. (2017). Structural disorder and induced folding within two cereal, ABA stress and ripening (ASR) proteins. Sci. Rep..

[23] Tompa P. (2002). Intrinsically unstructured proteins. Trends Biochem. Sci..

[24] Corrado G., Arena S., Araujo-Burgos T., Coppola M., Rocco M., Scaloni A., Rao R. (2016). The expression of the tomato prosystemin in tobacco induces alterations irrespective of its functional domain. Plant Cell Tissue Organ Cult..

[25] Corrado G., Alagna F., Rocco M., Renzone G., Varricchio P., Coppola V., Coppola M., Garonna A., Baldoni L., Scaloni A. (2012). Molecular interactions between the olive and the fruit fly *Bactrocera oleae*. BMC Plant Biol..

[26] Truppo E., Supuran C.T., Sandomenico A., Vullo D., Innocenti A., Di Fiore A., Alterio V., De Simone G., Monti S.M. (2012). Carbonic anhydrase VII is S-glutathionylated without loss of catalytic activity and affinity for sulfonamide inhibitors. Bioorg. Med. Chem. Lett..

[27] Pontiggia D., Ciarcianelli J., Salvi G., Cervone F., De Lorenzo G., Mattei B. (2015). Sensitive detection and measurement of oligogalacturonides in Arabidopsis. Front. Plant Sci..

[28] Di Lelio I., Varricchio P., Di Prisco G., Marinelli A., Lasco V., Caccia S., Casartelli M., Giordana B., Rao R., Gigliotti S. (2014). Functional analysis of an immune gene of *Spodoptera littoralis* by RNAi. J. Insect Physiol..

[29] Corrado G., Bovi P.D., Ciliento R., Gaudio L., Di Maro A., Aceto S., Lorito M., Rao R. (2005). Inducible expression of a *Phytolacca heterotepala* ribosome-inactivating protein leads to enhanced resistance against major fungal pathogens in tobacco. Phytopathology.

[30] Götz S., García-Gómez J.M., Terol J., Williams T.D., Nagaraj S.H., Nueda M.J., Robles M., Talón M., Dopazo J., Conesa A. (2008). High-throughput functional annotation and data mining with the Blast2GO suite. Nucleic Acids Res..

[31] Livak K.J., Schmittgen T.D. (2001). Analysis of relative gene expression data using real-time quantitative PCR and the 2^−ΔΔCT^ method. Methods.

[32] Underwood W. (2012). The plant cell wall: A dynamic barrier against pathogen invasion. Front. Plant Sci..

[33] Naoumkina M.A., Zhao Q., Gallego-Giraldo L., Dai X., Zhao P.X., Dixon R.A. (2010). Genome-wide analysis of phenylpropanoid defence pathways. Mol. Plant Pathol..

[34] Xu L., Zhu L., Tu L., Liu L., Yuan D., Jin L., Long L., Zhang X. (2011). Lignin metabolism has a central role in the resistance of cotton to the wilt fungus Verticillium dahliae as revealed by RNA-Seq-dependent transcriptional analysis and histochemistry. J. Exp. Bot..

[35] Leon-Reyes A., Spoel S.H., De Lange E.S., Abe H., Kobayashi M., Tsuda S., Millenaar F.F., Welschen R.A., Ritsema T., Pieterse C.M. (2009). Ethylene modulates the role of nonexpressor of pathogenesis-related genes1 in cross talk between salicylate and jasmonate signaling. Plant Physiol..

[36] Martínez-Medina A., Fernandez I., Lok G.B., Pozo M.J., Pieterse C.M., Van Wees S.C. (2017). Shifting from priming of salicylic acid-to jasmonic acid-regulated defences by Trichoderma protects tomato against the root knot nematode *Meloidogyne incognita*. New Phytol..

[37] Molinari S., Leonetti P. (2019). Bio-control agents activate plant immune response and prime susceptible tomato against root-knot nematodes. PLoS ONE.

[38] Albert M. (2013). Peptides as triggers of plant defence. J. Exp. Bot..

[39] Farrokhi N., Whitelegge J.P., Brusslan J.A. (2008). Plant peptides and peptidomics. Plant Biotechnol. J..

[40] Wang L., Einig E., Almeida-Trapp M., Albert M., Fliegmann J., Mithöfer A., Kalbacher H., Felix G. (2018). The systemin receptor SYR1 enhances resistance of tomato against herbivorous insects. Nat. Plants.

[41] Kim P.M., Sboner A., Xia Y., Gerstein M. (2008). The role of disorder in interaction networks: A structural analysis. Mol. Syst. Biol..

[42] Wallmann A., Kesten C. (2020). Common functions of disordered proteins across evolutionary distant organisms. Int. J. Mol. Sci..

[43] Cairns N.G., Pasternak M., Wachter A., Cobbett C.S., Meyer A.J. (2006). Maturation of Arabidopsis seeds is dependent on glutathione biosynthesis within the embryo. Plant Physiol..

[44] Gallé Á., Czékus Z., Bela K., Horváth E., Ördög A., Csiszár J., Poór P. (2019). Plant glutathione transferases and light. Front. Plant Sci..

[45] Gulyás Z., Boldizsár Á., Novák A., Szalai G., Pál M., Galiba G., Kocsy G. (2014). Central role of the flowering repressor ZCCT2 in the redox control of freezing tolerance and the initial development of flower primordia in wheat. BMC Plant Biol..

[46] Vernoux T., Wilson R.C., Seeley K.A., Reichheld J.-P., Muroy S., Brown S., Maughan S.C., Cobbett C.S., Van Montagu M., Inzé D. (2000). The root meristemless1/cadmium sensitive2 gene defines a glutathione-dependent pathway involved in initiation and maintenance of cell division during postembryonic root development. Plant Cell.

[47] Walling L.L. (2000). The myriad plant responses to herbivores. J. Plant Growth Regul..

[48] Walling L.L. (2009). Adaptive defense responses to pathogens and insects. Adv. Bot. Res..

[49] Camps M., Nichols A., Arkinstall S. (2000). Dual specificity phosphatases: A gene family for control of MAP kinase function. FASEB J..

[50] Gao X., Cox K.L., He P. (2014). Functions of calcium-dependent protein kinases in plant innate immunity. Plants.

[51] Liu Y., Huang X., Li M., He P., Zhang Y. (2016). Loss-of-function of Arabidopsis receptor-like kinase BIR 1 activates cell death and defense responses mediated by BAK 1 and SOBIR 1. New Phytol..

[52] Johnson K.L., Cassin A.M., Lonsdale A., Bacic A., Doblin M.S., Schultz C.J. (2017). Pipeline to identify hydroxyproline-rich glycoproteins. Plant Physiol..

[53] Pearce G., Ryan C.A. (2003). Systemic signaling in tomato plants for defense against herbivores: Isolation and characterization of three novel defense-signaling glycopeptide hormones coded in a single precursor gene. J. Biol. Chem..

[54] Ellinger D., Voigt C.A. (2014). Callose biosynthesis in Arabidopsis with a focus on pathogen response: What we have learned within the last decade. Ann. Bot..

[55] Voxeur A., Habrylo O., Guénin S., Miart F., Soulié M.-C., Rihouey C., Pau-Roblot C., Domon J.-M., Gutierrez L., Pelloux J. (2019). Oligogalacturonide production upon *Arabidopsis thaliana*–*Botrytis cinerea* interaction. Proc. Natl. Acad. Sci. USA.

[56] Hardham A.R., Jones D.A., Takemoto D. (2007). Cytoskeleton and cell wall function in penetration resistance. Curr. Opin. Plant Biol..

[57] Hardham A.R. (2013). Microtubules and biotic interactions. Plant J..

[58] Moral J., Montilla-Bascón G., Canales F.J., Rubiales D., Prats E. (2017). Cytoskeleton reorganization/disorganization is a key feature of induced inaccessibility for defence to successive pathogen attacks. Mol. Plant Pathol..

[59] Schmelzer E. (2002). Cell polarization, a crucial process in fungal defence. Trends Plant Sci..

[60] Janda M., Matoušková J., Burketová L., Valentová O. (2014). Interconnection between actin cytoskeleton and plant defense signaling. Plant Signal. Behav..

[61] Shi W., Zhang Y., Chen S., Polle A., Rennenberg H., Luo Z.B. (2019). Physiological and molecular mechanisms of heavy metal accumulation in nonmycorrhizal versus mycorrhizal plants. Plant Cell Environ..

[62] Tholl D. (2015). Biosynthesis and biological functions of terpenoids in plants. Biotechnol. Isoprenoids.

[63] Parizad S., Dizadji A., Habibi M.K., Winter S., Kalantari S., Movi S., Tendero C.L., Alonso G.L., Moratalla-Lopez N. (2019). The effects of geographical origin and virus infection on the saffron (*Crocus sativus* L.) quality. Food Chem..

[64] Bernards M.A., Båstrup-Spohr L., Schaller A. (2008). Phenylpropanoid metabolism induced by wounding and insect herbivory. Induced Plant Resistance to Herbivory.

[65] Gallie D.R. (2015). Appearance and elaboration of the ethylene receptor family during land plant evolution. Plant Mol. Biol..

[66] Ferrari S., Savatin D.V., Sicilia F., Gramegna G., Cervone F., De Lorenzo G. (2013). Oligogalacturonides: Plant damage-associated molecular patterns and regulators of growth and development. Front. Plant Sci..

[67] Bacete L., Mélida H., Miedes E., Molina A. (2018). Plant cell wall-mediated immunity: Cell wall changes trigger disease resistance responses. Plant J..

[68] Savatin D.V., Ferrari S., Sicilia F., De Lorenzo G. (2011). Oligogalacturonide-auxin antagonism does not require posttranscriptional gene silencing or stabilization of auxin response repressors in Arabidopsis. Plant Physiol..

[69] Qi L., Yan J., Li Y., Jiang H., Sun J., Chen Q., Li H., Chu J., Yan C., Sun X. (2012). *Arabidopsis thaliana* plants differentially modulate auxin biosynthesis and transport during defense responses to the necrotrophic pathogen *Alternaria brassicicola*. New Phytol..

[70] Cervone F., Hahn M.G., De Lorenzo G., Darvill A., Albersheim P. (1989). Host-pathogen interactions: XXXIII. A plant protein converts a fungal pathogenesis factor into an elicitor of plant defense responses. Plant Physiol..

[71] Orozco-Cardenas M., Ryan C.A. (1999). Hydrogen peroxide is generated systemically in plant leaves by wounding and systemin via the octadecanoid pathway. Proc. Natl. Acad. Sci. USA.

[72] Denoux C., Galletti R., Mammarella N., Gopalan S., Werck D., De Lorenzo G., Ferrari S., Ausubel F.M., Dewdney J. (2008). Activation of defense response pathways by OGs and Flg22 elicitors in Arabidopsis seedlings. Mol. Plant..

[73] Federici L., Di Matteo A., Fernandez-Recio J., Tsernoglou D., Cervone F. (2006). Polygalacturonase inhibiting proteins: Players in plant innate immunity?. Trends Plant Sci..

[74] Ferrari S., Galletti R., Denoux C., De Lorenzo G., Ausubel F.M., Dewdney J. (2007). Resistance to *Botrytis cinerea* induced in Arabidopsis by elicitors is independent of salicylic acid, ethylene, or jasmonate signaling but requires phytoalexin deficient3. Plant Physiol..

[75] Davidsson P., Broberg M., Kariola T., Sipari N., Pirhonen M., Palva E.T. (2017). Short oligogalacturonides induce pathogen resistance-associated gene expression in *Arabidopsis thaliana*. BMC Plant Biol..

[76] Montesano M., Kõiv V., Mäe A., Palva E.T. (2001). Novel receptor-like protein kinases induced by *Erwinia carotovora* and short oligogalacturonides in potato. Mol. Plant Pathol..

[77] Ridley B.L., O’Neill M.A., Mohnen D. (2001). Pectins: Structure, biosynthesis, and oligogalacturonide-related signaling. Phytochemistry.

[78] Simpson S., Ashford D., Harvey D., Bowles D. (1998). Short chain oligogalacturonides induce ethylene production and expression of the gene encoding aminocyclopropane 1-carboxylic acid oxidase in tomato plants. Glycobiology.

[79] Thain J., Gubb I., Wildon D. (1995). Depolarization of tomato leaf cells by oligogalacturonide elicitors. Plant Cell Environ..

[80] Weber J., Olsen O., Wegener C., Von Wettstein D. (1996). Digalacturonates from pectin degradation induce tissue responses against potato soft rot. Physiol. Mol. Plant Path..

[81] Li B., Meng X., Shan L., He P. (2016). Transcriptional regulation of pattern-triggered immunity in plants. Cell Host Microbe.

[82] Norman-Setterblad C., Vidal S., Palva E.T. (2000). Interacting signal pathways control defense gene expression in Arabidopsis in response to cell wall-degrading enzymes from *Erwinia carotovora*. Mol. Plant Microbe Interact..

