# Supplementary material for: Tomato Prosystemin Is Much More than a Simple Systemin Precursor
Source: Biology (Basel). 2022 Jan 13;11(1):124. doi: 10.3390/biology11010124 (PMC8772835; doi:10.3390/biology11010124)
Supplement: Supplementary file 1 [file biology-11-00124-s001.zip › Table_S1_new.pdf]

Table S1. Primer and their main features. Table reports primer name, sequence, amplicon length, name/gene symbol and identifier (ITAG annotation) used in the experimental procedures.

Primer	Sequence (5'→3')	Amplicon length (bp)	Name/Gene symbol	Accession Number
P11F1 Rw	CGCGCGCCATGGGAACTCCTTCATATGATATCA	560	ProSys$_{(1-178)}$	AAA34184.1
Del Rv	CGCGCGCTCGAGTTACTAAAGATCTTCTCGTACTAT AATTTTCTCAC			
GST Fw	AGATGAGACATTTGAAGGCCCT	106	GST	Solyc09g011500.2.1
GST Rv	TACCACCGCTCCCACCTTAT			
EF Fw Rt	CTCCATTGGGTCGTTTTGCT	101	elongation Factor 1 alpha	Solyc06g005060.2
EF Rv Rt	GGTCACCTTGGCACCAGTTG			
bZIP Fw	CGGCGTGTGGAAGATGAGAT	145	bZIP	Solyc01g090270.2.1
bZIP Rv	CCTCAAGGGCCTCTGTCATC			
PPR2 Fw	ATACCCAGAACGACCCGGTA	103	PPR2	Solyc01g110460.2.1
PPR2 Rv	CACCACCAGTCCCGATTCTC			
MLO3 Fw	CGGCACATTTAGCACCACAG	136	MLO3	Solyc06g010030.2.1
MLO3 Rv	GAGTAAGAAGAGCACGGCGA			
Crocetin Fw	GGAGTACCTGTCGTGGCTTT	83	Crocetin	Solyc12g098590.1.1
Crocetin Rv	ACTCCACTCTTCCACACATCT			
SRP Fw	CTTAGTGGACACAGCGAGCA	123	Stress-related protein	Solyc09g074930.2.1
SRP Rv	GATCTCCATGCCAAAACCGC			
DSP Fw	TCATCAGCCTCATCACCTCC	148	Dual specificity phosphatase 1	Solyc05g054700.2.1
DSP Rv	TGGAGTCAGACGGTGTTGAA			
Lap Fw	ATCTCAGGTTTCCTGGTGGAAGGA	99	leucine amino peptidase	Solyc00g187050.2
Lap Rv	AGTTGCTATGGCAGAGGCAGAG			
Mate Fw	ACCCATCAATGACACCCAAG	154	mate efflux protein	BI933305
Mate Rv	GGCATGTGGTATGGGATGTT			
PG Fw	TCAGCCCTTTTCCTTGTGCA	125	Polygalacturonase	Solyc08g082170.2.1
PG Rv	TGCGACCACCATCATTTCCA			
PcSys Fw	TCTGAATTTGTCTCCCGTTAGAA	119	Endogenous Prosystemin	Solyc05g051750.2.1
PcSys Rv	AGCCAAAAGAAAGGAAGCAATAC			
Sys Fw	GGGAGGGTGCACTAGAAATA	108	ProSystemin	Solyc05g051750.2.1
Sys Rv	TTGTCGAAACCGATGATACG			
Sysdel Fw	GAAGGAGATGATGATGCACAA	208	ProSystemin	Solyc05g051750.2.1
Sysdel Rv	CACCCTCCCCTTCCATCT			
