# Supplementary material for: Tomato Prosystemin Is Much More than a Simple Systemin Precursor
Source: Biology (Basel). 2022 Jan 13;11(1):124. doi: 10.3390/biology11010124 (PMC8772835; doi:10.3390/biology11010124)
Supplement: Supplementary file 1 [file biology-11-00124-s001.zip › Table_S2.pdf]

Table S2. Weight increase of *S. littoralis* larvae fed on leaves of $ProSys_{(1\text{-}178)}$ transgenic plants. The leaves employed were taken from control plants (C) and from two transgenic plant lines: $ProSys_{(1\text{-}178)}$ line 1 and 2, respectively. Average values ± standard deviations are reported for each sample for all experimental time points analyzed. For each time point, mean values denoted with different letters are significantly different (*One-way ANOVA: $P < 0.0001$ followed by Tukey's post-hoc multiple-comparison test).

Days of feeding	C<br>Larval weight (g)	$ProSys_{(1\text{-}178)}$ line 1<br>Larval weight (g)	$ProSys_{(1\text{-}178)}$ line 2<br>Larval weight (g)	F	P value
1	**0.0047** ± 0.0007 ^{a}	**0.0046** ± 0.0006 ^{a}	**0.0046** ± 0.0007 ^{a}	F (3. 124) = 1.584	0.1966
5	**0.0144** ± 0.0037^{a}	0.0131 ± 0.0027 ^{a}	**0.0101** ± 0.0016 ^{b}	F (3. 115) = 21.96	0.0001 *
10	**0.0226** ± 0.0091 ^{a}	**0.01642** ± 0.0054 ^{b}	**0.0159** ± 0.0039 ^{b}	F (3. 109) = 9.184	0.0001 *
15	**0.0964** ± 0.0341 ^{a}	**0.0266** ± 0.009 ^{b}	**0.0336** ± 0.036 ^{b}	F (3. 108) = 98.39	0.0001 *
20	**0.2763** ± 0.0913^{a}	**0.0545** ± 0.0134 ^{b}	**0.0455**± 0.0195^{b}	F (3. 98) = 144.1	0.0001 *
25	**0.9309** ± 0.0155 ^{a}	**0.08124** ± 0.0256 ^{b}	**0.0746** ± 0.0253 ^{b}	F (3. 91) = 2242	0.0001 *
