# Supplementary material for: Tomato Prosystemin Is Much More than a Simple Systemin Precursor
Source: Biology (Basel). 2022 Jan 13;11(1):124. doi: 10.3390/biology11010124 (PMC8772835; doi:10.3390/biology11010124)
Supplement: Supplementary file 1 [file biology-11-00124-s001.zip › Table_S3.pdf]

Table S3. Weight increase of *S. littoralis* larvae fed on leaves of tomato plants treated with ProSys$_{(1-178)}$recombinant protein. Averages ± standard deviations are reported for ProSys$_{(1-178)}$-treated and control (C) samples for all experimental time points. (Student's t-test: * *P* <0.01; ** *P* <0.0001).

Days of feeding	C Larval weight (g)	ProSys$_{(1-178)}$ Larval weight (g)	t	dF	*P*value
1	**0.0039** ± 0.0005	**0.0039** ± 0.0006	0.3865	62	0.7005
3	**0.0082** ± 0.0026	**0.004**8 ± 0.0015	6.373	62	0.0001 **
5	**0.0156** ± 0.0044	**0.0096** ± 0.0168	2.929	60	0.0599
7	**0.0192** ± 0.0089	**0.0084** ± 0.0029	5.918	56	0.0001 **
10	**0.0321** ± 0.0083	**0.0173** ± 0.0082	6.148	48	0.0001 **
12	**0.0556** ± 0.0121	**0.0143** ± 0.0067	11.87	42	0.0001 **
14	**0.0849**± 0.0321	**0.0455** ± 0.0587	2.926	28	0.0067*
