# Supplementary figures and images for: Tomato Prosystemin Is Much More than a Simple Systemin Precursor
Source: Biology (Basel). 2022 Jan 13;11(1):124. doi: 10.3390/biology11010124 (PMC8772835; doi:10.3390/biology11010124)

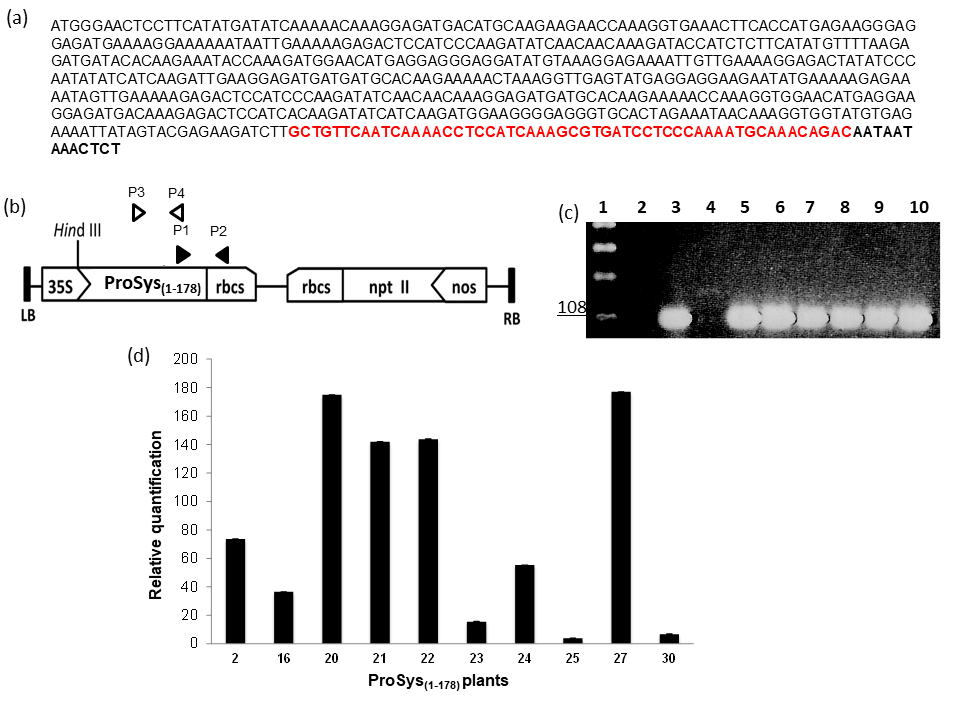

Supplement: Supplementary file 1 [file biology-11-00124-s001.zip › Figure_S1_new.tif]

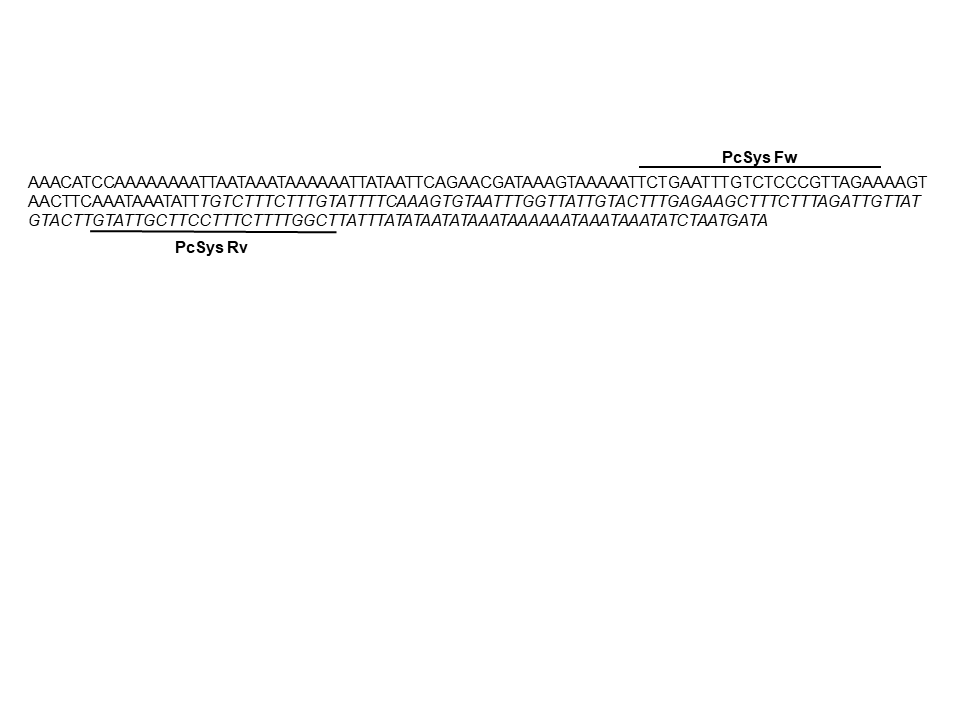

Supplement: Supplementary file 1 [file biology-11-00124-s001.zip › Figure_S2_new.tif]

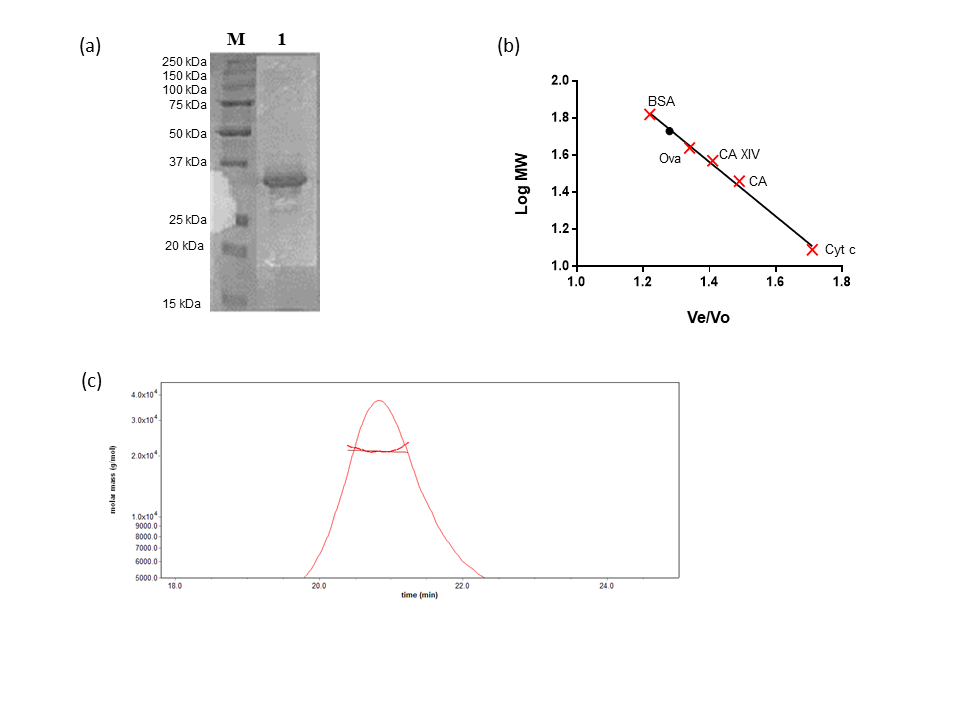

Supplement: Supplementary file 1 [file biology-11-00124-s001.zip › Figure_S3_new.tif]

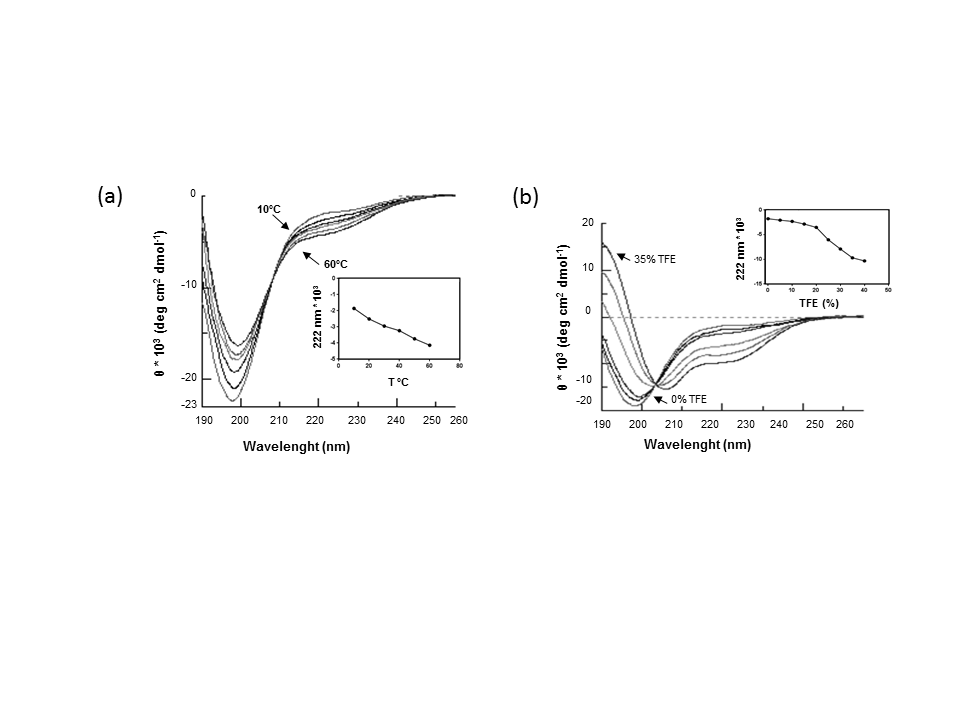

Supplement: Supplementary file 1 [file biology-11-00124-s001.zip › Figure_S4_new.tif]

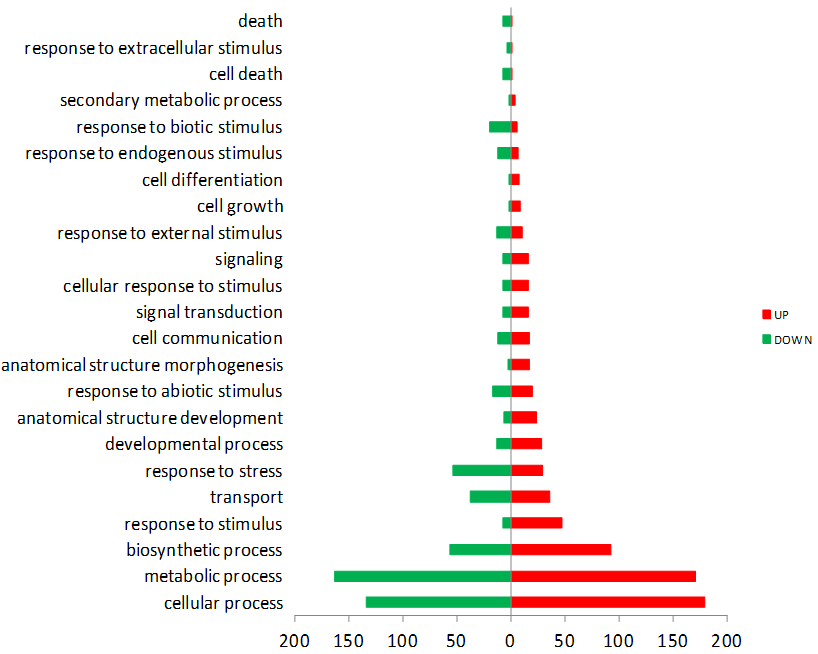

Supplement: Supplementary file 1 [file biology-11-00124-s001.zip › Figure_S5_new.tif]

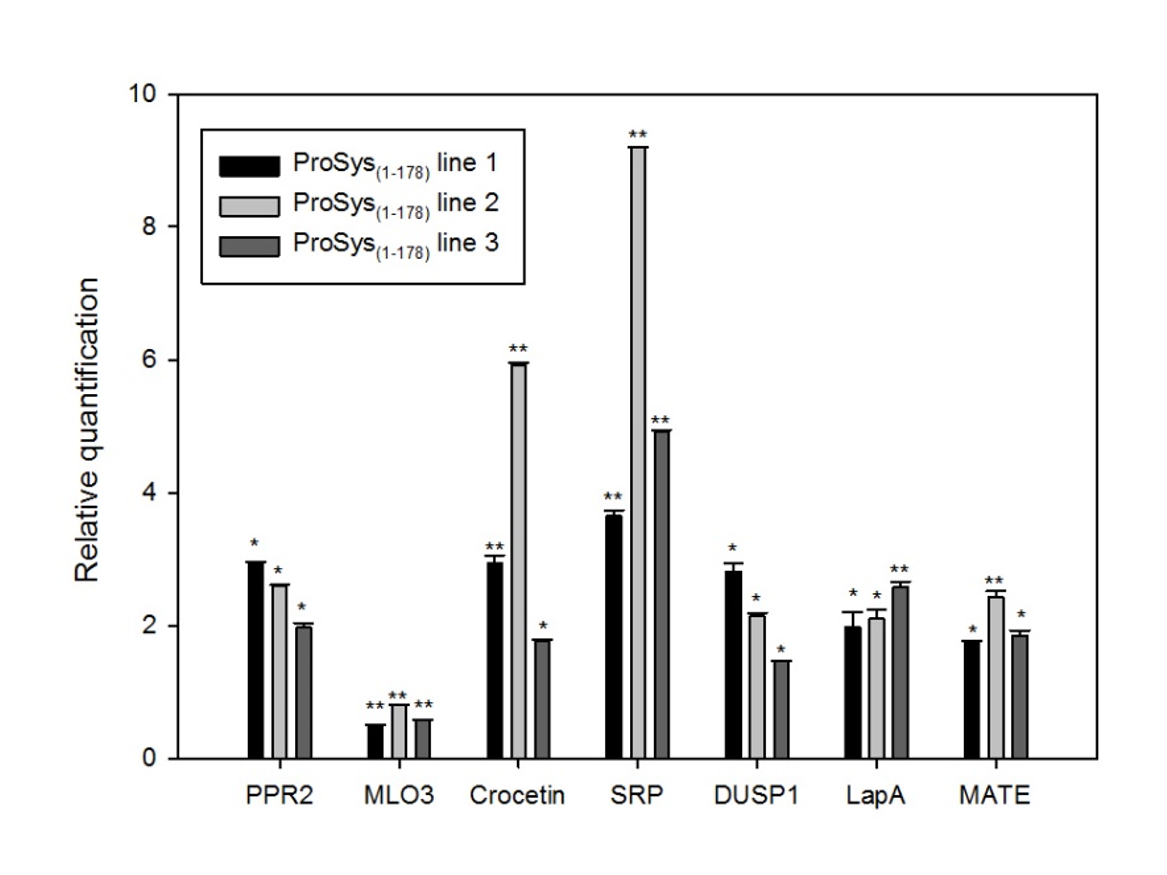

Supplement: Supplementary file 1 [file biology-11-00124-s001.zip › Figure_S6_new.tif]

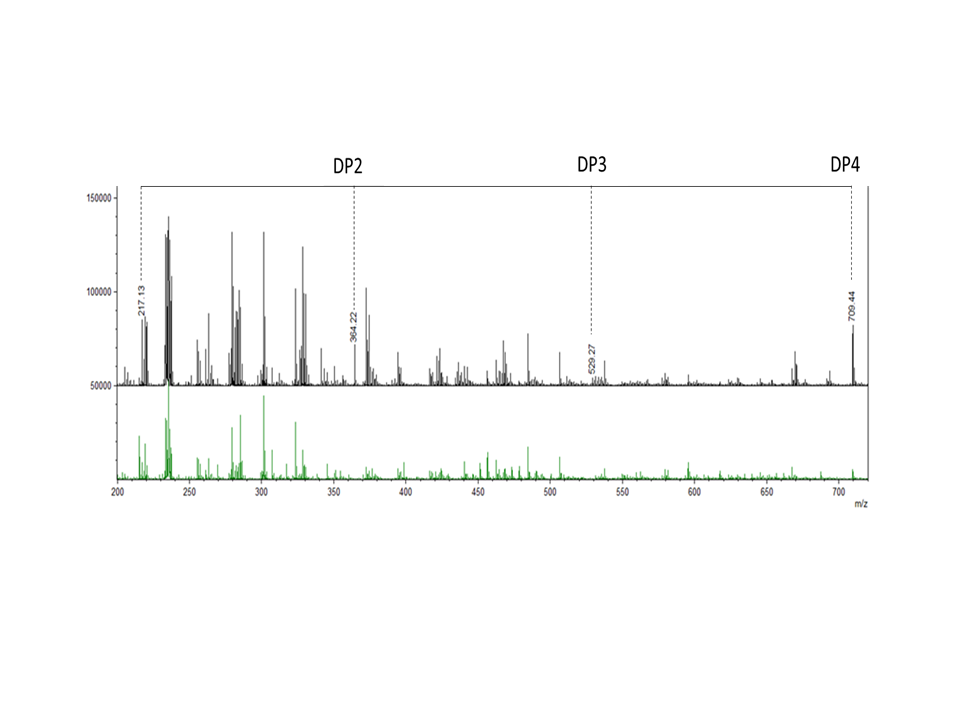

Supplement: Supplementary file 1 [file biology-11-00124-s001.zip › Figure_S7_new.tif]
